# Identification of neurotoxic cytokines by profiling Alzheimer’s disease tissues and neuron culture viability screening

**DOI:** 10.1038/srep16622

**Published:** 2015-11-13

**Authors:** Levi B. Wood, Ashley R. Winslow, Elizabeth A. Proctor, Declan McGuone, Daniel A. Mordes, Matthew P. Frosch, Bradley T. Hyman, Douglas A. Lauffenburger, Kevin M. Haigis

**Affiliations:** 1 Cancer Research Institute, Beth Israel Deaconess Cancer Center and Department of Medicine, Harvard Medical School, Boston, MA 02215, USA; 2 Department of Neurology, Massachusetts General Hospital and Mass General Institute for Neurodegenerative Disease, Charlestown, MA 02129, USA; 3 Department of Biological Engineering, Massachusetts Institute of Technology, Cambridge, MA 02139, USA; 4 James Homer Wright Pathology Laboratories, Massachusetts General Hospital and Department of Pathology, Harvard Medical School, Charlestown, MA 02129, USA; 5 C.S. Kubik Laboratory for Neuropathology, Massachusetts General Hospital and Department of Pathology, Harvard Medical School, Boston, MA 02114, USA

## Abstract

Alzheimer’s disease (AD) therapeutics based on the amyloid hypothesis have shown minimal efficacy in patients, suggesting that the activity of amyloid beta (Aβ) represents only one aspect of AD pathogenesis. Since neuroinflammation is thought to play an important role in AD, we hypothesized that cytokines may play a direct role in promoting neuronal death. Here, we profiled cytokine expression in a small cohort of human AD and control brain tissues. We identified AD-associated cytokines using partial least squares regression to correlate cytokine expression with quantified pathologic disease state and then used neuron cultures to test whether cytokines up-regulated in AD tissues could affect neuronal viability. This analysis identified cytokines that were associated with the pathological severity. Of the top correlates, only TNF-α reduced viability in neuron culture when applied alone. VEGF also reduced viability when applied together with Aβ, which was surprising because VEGF has been viewed as a neuro-protective protein. We found that this synthetic pro-death effect of VEGF in the context of Aβ was commensurate with VEGFR-dependent changes in multiple signaling pathways that govern cell fate. Our findings suggest that profiling of tissues combined with a culture-based screening approach can successfully identify new mechanisms driving neuronal death.

Alzheimer’s disease (AD) afflicts more than 30 million people worldwide. In the United States, due to an aging population and the lack of an effective therapy, AD is the only disease out of the six leading causes of death that featured a sharply increasing death rate during the last decade^1^. AD is characterized pathologically by the progressive appearance of senile plaques composed of amyloid beta (Aβ), followed by microglial and astrocytic immune responses^2^,^3^, formation of neurofibrillary tangles (NFTs), neuronal dystrophy, and neuronal death^4^. Despite the clear relevance of Aβ accumulation as an early marker of AD, clinical trials aimed at reducing Aβ burden by inhibiting cleavage of the amyloid precursor protein^5^ or via antibodies targeting Aβ have not been successful in slowing disease progression. Moreover, it has recently been reported that some mismatch individuals exhibit unusually high levels of Aβ accumulation in the brain without suffering significant cognitive decline or neuronal loss^6^. One important difference between mismatch and AD brains is that the mismatches exhibit a reduced level of neuroinflammation.

Glia serve the dual roles of acting as the primary immune system in the brain^3^,^7^ and regulating homeostasis of the tissue microenvironment^8^,^9^. Microglia initiate the inflammatory response in AD by migrating to surround Aβ plaques^10^. Fibrillar Aβ, found in plaques, is known to stimulate microglial secretion of pro-inflammatory cytokines, including IL-1, IL-6, and TNF-α^11^,^12^. Moreover, cytokines expressed by microglia (*e.g.*, MIP-1α, MCP-1) have been shown to stimulate astrocyte chemotaxis^13^, leading to astrocyte envelopment of the plaques. Whether these microglial and astrocytic responses are protective or deleterious has been a matter of debate. One line of thought is that microglial and astrocytic responses reflect a protective immune function aimed at sequestering and degrading plaques^14^. There is mounting evidence, however, that glial responses to secreted cytokines and Aβ contribute to AD pathogenesis by producing factors, such as nitric oxygen synthase, that contribute to neuronal death^15^,^16^. Moreover, certain cytokines, such as TNF-α, IFN-γ, and IL-6, have been implicated in neuronal death^17^,^18^,^19^ and IL-6 has been reported to up-regulate amyloid precursor protein synthesis and processing^20^, thereby accelerating plaque formation and disease progression.

We hypothesized that cytokines may directly contribute to neuronal death in AD. By extension, certain cytokines may represent previously unappreciated therapeutic targets. In this work, we analyzed cytokine concentration in a small cohort of postmortem human tissue samples to identify a profile of AD-associated cytokines that we used as a hypothesis generating tool for use in a neuron culture viability screen. By analyzing tissues from multiple regions of each brain, we were able to exploit the spatio-temporal nature of AD progression to identify cytokines that were most strongly associated in the most degenerated tissues. Our analysis identified vascular endothelial growth factor (VEGF), a cytokine that has been considered to be neurotrophic^21^,^22^, as the strongest correlate with the most severe AD pathology. Though our analysis was based on a small cohort, a screen of the top three cytokine correlates (VEGF, TNF-α, and IL-5) in primary cultures revealed that TNF-α reduced neuronal viability when applied either alone or together with Aβ. Surprisingly, VEGF also reduced viability, but only in the presence of Aβ. The effect of VEGF was commensurate with a broad decrease in pro-survival signaling and could be abrogated by co-application of a VEGFR1/2 inhibitor or brain-derived neurotrophic factor (BDNF). These results suggest that this pathway could be a target for AD therapy. More broadly, our findings suggest that *in vitro* screening based on proteomic analysis of primary tissues represents a viable methodology for identifying neurotoxic factors in AD.

## Results

### Multivariate regression identifies a profile of cytokines dysregulated in AD

**Figure 1:**
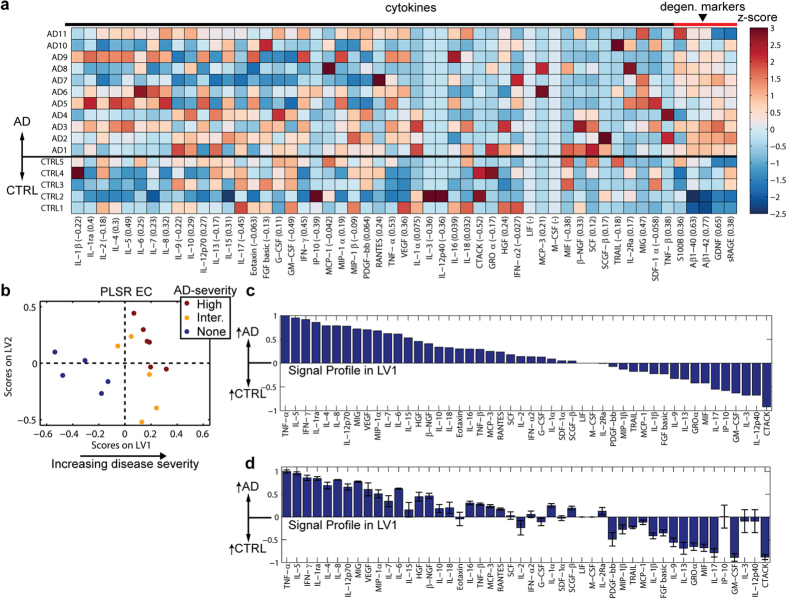
Computational modeling of cytokine protein expression in primary brain samples from AD patients. (**a**) Heat map of *z*-scored cytokine (black bar) and neurodegeneration signal (red bar) data measured from the EC of each subject using Bio-Plex analysis. Values in parentheses are the Pearson’s correlation coefficients relating each signal to ABC score. (**b**) A PLSR model constructed from the cytokine dataset regressed against “ABC” AD severity^26^. The model identifies a latent variable (LV1) that scores subjects based on cytokine protein expression measurements and predicts disease severity. LV2 is related to variation that is not connected to disease severity, perhaps genetic or environmental differences. LV1 and LV2 account for approximately 18% and 16% of the dataset variation, respectively. (**c**) LV1 is composed of a profile of cytokines that are elevated in either AD (positive) or control subjects (negative) and is able to predict disease severity. In a leave-one-out cross validation, the model predicts the true ABC value with a correlation coefficient *R* = 0.83 using the first two LVs (Supplementary Table S3). (**d**) Variation in contribution of each individual signal to LV1 for each of the 16 computational models generated in a leave-one-out cross validation (mean ± SD across LV1 generated for all models in the cross validation).

To gain insight into the state of the cytokine signaling network in AD, we performed high-throughput screening of cytokine protein concentrations on tissues collected at the ADRC brain bank between 10/18/2011 and 6/7/2012. We began by analyzing the entorhinal cortices (ECs) of AD brains (*N* = 11) and non-AD controls (*N* = 5), since the EC is the earliest and most profoundly impacted brain region in AD patients^23^. We analyzed pathology reports and assessed disease severity in terms of Braak stage^23^, Thal phase for Aβ plaques^24^, and CERAD score for neuritic plaques^25^ (Supplementary Table S1), which we combined to compute a composite “ABC” AD severity score^26^ (Supplementary Table S2). We then quantified 48 cytokines in each sample using Bio-Plex (Bio-Rad) (Fig. 1a). We also measured 5 biomarkers of neurodegenerative disease using the same technology (Millipore). All of these biomarkers were positively correlated with disease demonstrating that Bio-Plex technology is able to measure relevant proteins from postmortem tissues.

To account for the multivariate nature of the cytokine/AD relationship, we turned to a multivariate regression modeling approach, partial least squares regression (PLSR), that exploits highly multivariate datasets to distinguish signaling changes that correlate with disease severity score from unrelated noise in the measurements^27^,^28^,^29^,^30^. While this approach has rarely been applied to *in vivo* systems, we have successfully used it to identify signaling mechanisms related to TNF-α induced apoptosis in the mouse intestinal epithelium^31^. The regression analysis was able to separate subjects using a profile called a latent variable (LV) that correlated cytokine expression with pathological severity (Fig. 1b). The variable, LV1 (Fig. 1c), was composed of a collection of cytokines that positively or negatively correlated with ABC score in AD patients. Our regression revealed that, rather than isolated changes in cytokine signaling in AD, there was a widespread change in the intercellular signaling network.

As a check for over-fitting, we used a leave-one-out cross validation^30^ to verify that the regression model was able to predict the ABC severity of samples not used for generating the model. In turn, we left each sample out during the model generation and then predicted ABC score using the model generated from the remaining data points. Comparison of the model prediction with the true value produced a correlation coefficient of *R* **= **0.83 (Supplementary Table S3) and most of the cytokines showed low-to-moderate variability between each of the 16 models (Fig. 1d). This validation demonstrated that the regression model was a good predictor of pathological severity and that the involvement of each cytokine in LV1 was not the result of a contribution by any single sample. Repeating the PLSR analysis using only the A, B, or C score as the phenotypic variable yielded similar results (Supplementary Fig. S1), suggesting that the three methods for pathological scoring likely reflect similar changes in cytokine signaling.

While age distributions were similar between CTRL and AD groups (Supplementary Fig. S2a), a potential concern with our analysis was that the samples in the CTRL group were mostly from males, while the samples from the AD group were mostly from females (Supplementary Table S2). As such, our PLSR analysis may have identified a LV that merely discriminated between individuals of different gender. To check whether the cytokines in LV1 (Fig. 1c) were identified based on gender differences, we individually plotted EC measurements from the top 9 LV1 cytokines vs. gender and disease group (Supplementary Fig. S2b). Inspecting these top correlates does not indicate a consistent bias associated with gender, but also does not provide sufficient statistical power to indicate whether any one signal is statistically significant. Our goal, however, is to identify whether there is any bias based on the data points plotted in a multivariate model space, and by extension whether the model (i.e., LV1) distinguishes samples based on gender. To answer this question, we plotted the scores for the EC model and labeled them with AD-severity and gender, and added 95% confidence intervals for the CTRL and AD groups in the scores space (Supplementary Fig. S2c). The scores plots do not suggest a gender-associated bias in the model. Furthermore, plotting the scores along LV1 for each disease/gender group suggests that the AD male and female groups are not significantly different, while each of these groups is significantly different from the male CTRL group (Supplementary Fig. S2d).

### Gene expression profile reproduces changes in published data, but does not identify key changes in protein expression

Because our specimen cohort was relatively small and gender imbalanced (Supplementary Table S2), we wanted to gain confidence that PLSR analysis applied to our cohort could identify a cytokine profile that was consistent with previously published data. Our interest is in identifying *proteins* that may be directly harmful to neurons. Nevertheless, we decided to use gene expression as the basis for comparison, since published gene expression data is publically available, and these data encompass the genes for all of the proteins used in our Bio-Plex analysis.

**Figure 2:**
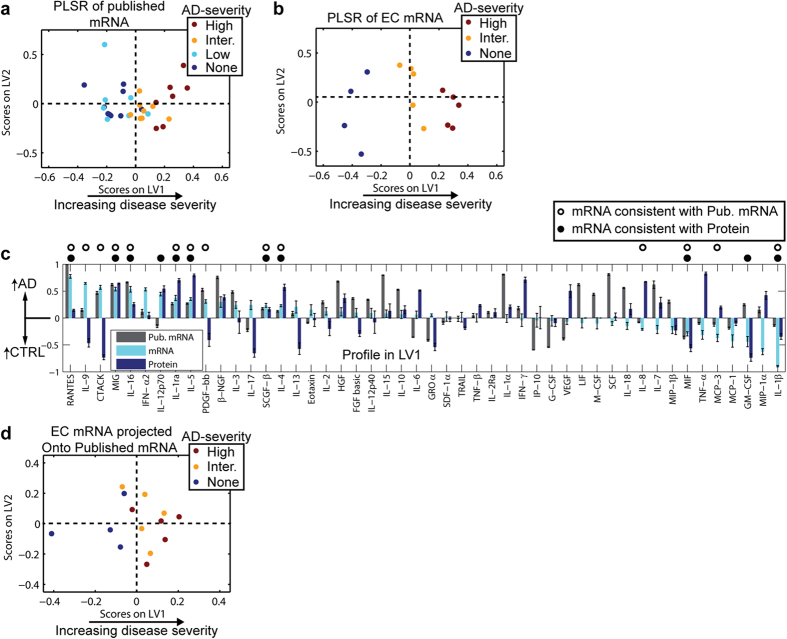
PLSR analysis of gene expression from our cohort is similar to a published dataset, but does not identify several important differences observed in the protein dataset. (**a**) PLSR of published hippocampal CA1 gene expression dataset^32^ was able to predict severity score with a correlation coefficient of R = 0.74 in a LOOCV. IL-12p35 was used as a surrogate for IL-12p70 in this analysis. LV1 is shown in Supplementary Fig. S3a. (**b**) PLSR analysis of gene expression from our EC tissues (Supplementary Fig. S3b) produced a model with ABC-score prediction capability (*R* = 0.85 in a LOOCV) that was similar to our Bio-Plex protein analysis (Fig. 1). LV1 is shown in Supplementary Fig. S3c. (**c**) Mean ± standard deviation of signals in LV1 for each model from the published mRNA, EC mRNA, and EC protein datasets. Plotting the LV1s together revealed many similarities between our EC mRNA data and the published dataset. Signals are highlighted to be consistent between the EC mRNA model and either the published mRNA model or the Bio-Plex EC model if the sign of the signal is the same for both models and it has a coefficient of variation < 0.25 for both models. (**d**) A PLSR model built on the top and bottom 5 correlates from the published dataset was able to distinguish AD vs. control cases, and predict ABC severity of our EC samples based on Quantigene-measured gene expression with *R* = 0.76.

We reasoned that gene expression differences from an available hippocampus microarray dataset may correlate with differences in the EC since the hippocampus is adjacent to the EC and is affected by NFTs just after the EC according to Braak stage^23^. The dataset^32^ consisted of microarray analysis of the CA1 region of the hippocampus (9 control and 22 AD cases), and was recently used for gene ontology (GO) analysis^33^. To compare against our protein dataset, we extracted expression values for the genes corresponding to our Bio-Plex cytokine panels. Similar to our ABC analysis, the authors quantified disease severity (*i.e.*, none, low, moderate, and high) based on MiniMental State Examination score, NFT quantification, amyloid pathology, and Braak stage. We converted these severity scores to numerical values (0–3, respectively), and used PLSR to regress the gene expression data against these scores. The resultant profile along LV1 segregated controls (none) and low cases on the left, intermediate cases in the middle, and advanced AD cases to the right in the scores plot (Fig. 2a and Supplementary Fig. S3a). The regression model produced *R* = 0.74 in a LOOCV (Supplementary Table S3).

To directly compare against our samples against the published dataset, we next used a Quantigene assay (Affymetrix) to quantify gene expression in our tissue samples (Supplementary Fig. S3b). We used PLSR to regress the Quantigene data against the ABC score for each sample, generating a profile that substantially overlapped with the profile obtained for the published dataset (Fig. 2b and Supplementary Fig. S3c). To assess similarity between the published mRNA dataset, our Affymetrix analysis, and our Bio-Plex analysis, we plotted mean and standard deviation for each signal in each model using a LOOCV (Fig. 2c). Signals possessing with same sign and with coefficients of variation <0.25 were labeled as consistent between datasets. Of the top 5 and bottom 5 correlates in the profile from our samples, 7 of them were consistent with the profile obtained for the published dataset (Fig. 2c). To further demonstrate the similarity between the published dataset and ours, we generated a PLSR model from the top and bottom 5 correlates from the published dataset, which are most likely to be robust in distinguishing samples based on gene expression. The result was that the new PLSR model was able to distinguish severity of our samples, with control cases clustered on the left, intermediate cases clustered toward the origin, and high-severity AD cases clustered toward the right (Fig. 2d). The model predicted the true ABC severity scores of our samples with a correlation coefficient *R* = 0.76. Together, these results demonstrate that changes observed in our small cohort reflect many of the same changes found in an independently-derived dataset with larger sample size.

While the gene expression datasets shared many similarities in their LV1 profiles, we noted that there were important differences between the gene expression and protein analysis for our samples. The protein profile (Figs 1c and 2c) showed correlated up-regulation of known AD-associated cytokines, including TNF-α^17^,^34^, and VEGF^35^, which were not up-regulated in AD based on either gene expression datasets (Fig. 2c). Since protein, rather than gene expression, governs cellular function, these differences suggest that gene expression may fail to identify important changes in protein concentration that drive disease.

### Cytokine profile is region-specific

**Figure 3:**
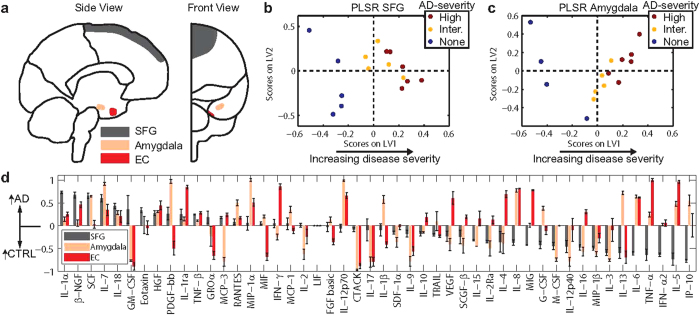
The cytokine profile distinguishing AD and control tissues varies spatially. (**a**) AD progresses spatio-temporally beginning in the EC, then into the limbic regions (including the amygdala), and finally reaching the SFG. (**b**) A PLSR model constructed from the cytokines measured from the SFG of the same control and AD subjects analyzed in Fig. 1. Data and LV1 are shown in Supplementary Fig. S4a,b. (**c**) A PLSR model constructed from the cytokines measured from the amygdala of the same control and AD subjects analyzed in Fig. 1. Data and LV1 are shown in Supplementary Fig. S4c,d. (**d**)) Mean ± standard deviation of signals in LV1 for each model from the SFG, amygdala, and EC (in ascending order of pathological severity) plotted side-by-side. The signals are sorted in descending order of LV1 from the SFG. The signals correlating most strongly with AD are different in the different brain regions.

While our PLSR analysis was able to identify a cytokine signature that could reliably discriminate between AD patients and controls, the biological relevance of any given cytokine in the signature was unclear because the EC of AD patients differs so significantly from normal brain in terms of, for example, cellular representation. AD does not strike all brain regions equally and simultaneously, but rather progresses through the brain, starting in the EC and spreading outwardly to the limbic system (including the hippocampus and amygdala) and then the cerebral cortex (Fig. 3a)^23^,^36^. We reasoned, therefore, that different regions of the same brain represent different stages of pathological severity. To characterize how signaling differs in tissues that are relatively mildly and moderately degenerated, we analyzed cytokine protein expression in the amygdala and superior frontal gyrus (SFG) (Supplementary Fig. S4), which first develop with NFTs at Braak stages III–IV and V–VI, respectively^23^. As with the EC, PLSR modeling identified LVs that distinguished AD patients from non-AD controls (Fig. 3b,c).

Comparative analyses of the LOOCV PLSR models from SFG, amygdala, and EC demonstrated that the SFG and amygdala were more consistent with one another than with the strongly affected EC. For example, some of the strongest cytokine correlates in the EC (*e.g.*, TNF-α, IFN-γ) were not as strong in the SFG and amygdala, while other cytokines (*e.g.*, IL-7) were stronger in the SFG and amygdala than in the EC (Fig. 3d). Interestingly, this analysis revealed that IL-1α/β expression was correlated with early and intermediate disease, but not late disease (Fig. 3d), which is consistent with prior work^37^. Altogether, this analysis suggested that the cytokine signaling network evolves as AD pathology progresses throughout the brain.

### Identification of cytokines linked to pathological progression

Our analysis of cytokine expression in different brain regions revealed that the cytokine signaling network evolves with AD severity. We reasoned that a PLSR analysis that is able to take into account the changes in cytokine expression with increasing pathological severity on a patient-by-patient basis would be more likely to identify cytokines that play a causal role in the disease.

**Figure 4:**
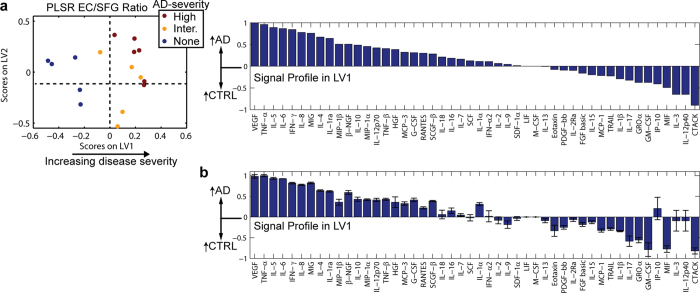
Ratio analysis identifies cytokines linked to advanced AD pathology. (**a**) PLSR analysis of the EC/SFG ratio for each cytokine in each patient. This analysis identified VEGF, TNF-α, and IL-5 as the three cytokines most strongly correlated with increased pathological severity. (**b**) Variation in contribution of each individual signal’s EC/SFG ratio to LV1 for each of the 16 computational models generated in a leave-one-out cross validation (mean ± SD across LV1 generated for all models in the cross validation).

To identify the cytokines that were up-regulated in the EC (advanced pathology) relative to the SFG (relatively mild pathology), we normalized the EC measurement for each cytokine by the corresponding measurement in the SFG from the same case and again performed PLSR (Fig. 4a). The result was a new profile that again showed low-to-moderate variability in each of the cytokines (Fig. 4b), and marginally improved the ABC prediction capability of the regression model (*R* = 0.86 in a LOOCV) compared with the model generated for the EC alone (Fig. 1c, Supplementary Table S3). To eliminate noise due to some signal measurements in the SFG reading at, or near, zero, we added a constant to each signal in the SFG (see Methods). The resulting model was insensitive to the magnitude of the constant added (Supplementary Fig. S5a). Like with the EC analysis, the scores plotted along LV1 did not suggest a gender-associated bias (Supplementary Fig. S5c,d). Importantly, the strongest cytokine signals identified by the ratio model differed from those in the regional models; VEGF, IL-6, IL-10, and MIP-1β were among the cytokines that were more strongly correlated with AD in the ratio model (Fig. 4a).

The top three cytokines from this analysis were VEGF, TNF-α, and IL-5. Of these, both TNF-α and VEGF have been connected to AD pathology. Only TNF-α has been implicated as a driver of neuronal death, however, while VEGF and IL-5 have been shown to be neurotrophic (see Supplementary Results).

### Profile-identified cytokines promote neuronal death in culture

**Figure 5:**
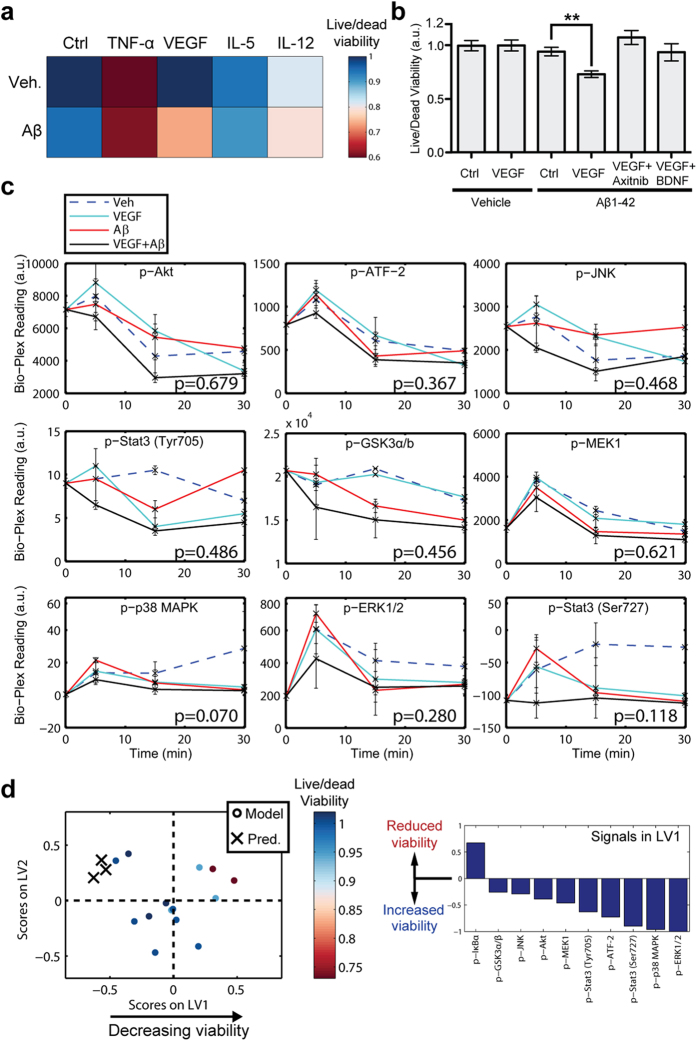
Aβ and VEGF cooperate to affect neuronal viability and phospho-protein signaling. (**a**) The top three up-regulated cytokines in the EC/SFG ratio model and IL-12p70 were applied to primary mouse neuron cultures together with a physiologic level of recombinant Aβ1–42 (50 nM)^56^ or vehicle (0.001% w/v NH_4_OH) for 3 days, then assessed for viability using Live/Dead staining (Supplementary Fig. S6a). The color-map indicates mean viability for each condition. This analysis revealed that TNF-α and IL-12p70 reduced neuronal viability when applied alone, while VEGF only did so in the presence of Aβ1–42. (**b**) VEGF significantly reduced neuron viability, but only in the context of Aβ1–42. This effect could be abrogated either by applying VEGF and Aβ1–42 together with the VEGFR1/2 inhibitor Axitnib or with the trophic factor BDNF. (Bars represent mean ± SE for each condition. Left-to-right: *N* = 192, 120, 190, 216, 48, 48; **p < 0.0001; two-sided Wilcoxon Rank Sum test). (**c**) Kinetic analysis of key phospho-protein signaling nodes in primary neuron cultures. Cultures were conditioned in 6-well plates with vehicle, Aβ1–42 (50 nM), and/or VEGF (100 ng/mL), then lysed at 0, 5, 15, and 30 min post-treatment (mean ± SE, *N* = 2, p-values were computed using two-tailed t-test comparing the Aβ and VEGF + Aβ conditions at the 5 min time point, no correction was used for multiple hypothesis testing). (**d**) PLSR modeling of 5 min time point phospho-protein signaling in cells treated with Aβ and/or VEGF, and Axitnib. Signals were regressed against mean viability values for each condition from Fig. 5b. LV1 identifies phospho-protein signals that correlate with neuronal death in cells co-treated with Aβ and VEGF. Phosphorylation of IκBα positively correlated with neuronal death, while a variety of signals were negatively correlated with this phenotype. X’s represent the predicted outcome along LV1 in cells treated with Aβ, VEGF, and BDNF, based on signals measured from this treatment group. The X’s cluster with samples treated with vehicle, VEGF alone, or VEGF + Aβ + Axitnib, correctly predicting that BDNF would reverse the pro-death synthetic phenotype associated with Aβ and VEGF.

To determine whether the cytokines identified from our regression profile could potentially drive AD pathogenesis, we assessed viability of primary mouse neuron cultures treated with combinations of Aβ, the top three up-regulated cytokines from the EC/SFG ratio analysis (VEGF, TNF-α IL-5), and IL-12p70, which is among the top 11 cytokines identified in all regions Fig. 1c and Supplementary Fig. S4b,d and has been implicated in AD^38^. We found that TNF-α and IL-12p70 both reduced neuronal viability in the presence or absence of Aβ (Fig. 5a and Supplementary Fig S6a), which is consistent with prior work implicating them in AD pathogenesis^34^,^38^. We did not detect significant activity associated with IL-5 in this assay, but, surprisingly, we found that VEGF significantly reduced viability when applied together with Aβ (Fig. 5a). The synthetic pro-death effect of VEGF could be abrogated using the small molecule VEGFR1/2 inhibitor, Axitinib (Fig. 5b). The ability of VEGF to promote neuronal death was unexpected, since VEGF has widely been reported to be neurotrophic^39^ and involved in neuronal development^40^.

Because VEGF has not previously been implicated in promoting AD progression, we sought to identify a downstream signaling mechanism that is responsible for the synthetic neuronal cell death phenotype. To this end, we used Bio-Plex analysis to measure the activation state of key phospho-protein signaling nodes downstream of receptor tyrosine kinases (Supplementary Fig. S6b) that might be responsible for cell fate in cells treated acutely with VEGF and/or Aβ (Fig. 5c). A similar analysis was not possible in primary human samples because phosphorylation signals are not retained in post-mortem tissue. While none of the differences between the Aβ and VEGF+Aβ conditions were significant in this initial analysis, the temporal signaling curves suggested that multiple signals may be reduced in the VEGF+Aβ at the 5 min time point, including, Erk, p38 MAPK, and Stat3 (Ser727). We next used PLSR on signaling data acquired from a separate experiment to identify signaling nodes at the 5 min time point correlated with reduced neuronal viability by regressing against mean viability values from each treatment condition (Fig. 5d). This analysis revealed IκBα, a member of the NFκB pathway, as the only signal positively correlated with neuronal death after exposure to VEGF and Aβ. There were several pro-survival signals, some of which were in the MAPK pathway, that were negatively correlated with neuronal death. Based on this observation, we hypothesized that an extracellular signal that could broadly activate these pro-survival signals independently of VEGFR would overcome the pro-death effects of Aβ and VEGF.

To induce phosphorylation of these down-regulated signals, we used BDNF, a neurotrophic factor that is down-regulated in AD^41^ and has previously been shown to promote neuron survival via MAPK signaling in neuron cultures^42^. We found that BDNF, could induce phosphorylation of many of the signals that were negatively correlated with neuronal death in cells treated with Aβ and VEGF (Supplementary Fig. S6c). Moreover, when we collected signaling data from cells treated with Aβ, VEGF, and BDNF, our PLSR model predicted that the cells would phenocopy those treated with vehicle alone (Fig. 5d). Consistent with this PLSR model prediction, BDNF restored viability in neurons treated with Aβ and VEGF (Fig. 5b).

## Discussion

The lack of efficacy of drugs targeting Aβ suggests that other aspects of AD pathogenesis contribute to neuronal death. We hypothesized that dysregulated expression of cytokines secondary to glial activation during neuroinflammation contributes to AD progression by promoting neuronal death. The body of knowledge relating cytokine expression/function to neuronal homeostasis and neurodegenerative disease is extensive, but lacking a cohesive investigation to identify specific cytokines promoting AD pathogenesis (Supplementary Results). Our methodology was founded upon the use of a multivariate regression analysis of cytokine expression in human brain tissues against pathological severity to suggest hypotheses about potentially neurotoxic cytokines. We then tested hypotheses suggested by the regression analysis in neuron cultures to identify whether specific cytokines could promote neuronal death.

A potentially confounding issue with our tissue analysis was that the samples were drawn from a genetically diverse population that died of unknown, and potentially multiple, causes. Despite these potential sources of noise in the dataset and despite the small sample size (11 AD cases, and 5 controls), the EC PLSR model prediction of ABC severity strongly correlated with the true pathological severity assessment in a leave-one-out cross validation (*R* = 0.83). We view the ability of the PLSR model to predict pathological severity in samples that were not included in the model — despite potentially confounding sources of variation in the data — as an important validation of our approach. We also computed the mean and standard deviation for each of the signals, in the EC and EC/SFG ratio models, to quantify how much the involvement of each signal varied as each sample was left out of the analysis. The result was that most signals had low-to-moderate variation between models (Figs 1d and 4b).

The measurement of protein concentration is a key feature of our analysis — we found that differential gene expression analysis (Fig. 2c) did not identify important changes in protein concentrations measured from the same collection of EC samples (Fig. 1c), including changes in TNF-α and VEGF, which have previously been connected to AD pathology^17^,^34^,^35^. Our observed differences between gene and protein expression may be due to the multiple steps involved in protein synthesis and regulation post-gene expression. Since cell function is governed by protein expression, rather than gene expression, these data suggest that analysis based on protein expression measurement may be more likely to reveal mechanisms of disease.

The comparison between gene expression in our samples and the gene expression in the published dataset indicates that our small cohort may reflect a “true” profile as measured by other authors, despite a gender imbalance between AD and CTRL groups (Supplementary Table S2). Furthermore, plotting the scores for the EC and ratio models for each gender/disease does not indicate a gender bias along LV1. Nevertheless, we emphasize here that our protein analysis represents a hypothesis-generating tool based on a preliminary dataset, rather than a validated characterization of cytokine signaling in AD.

We analyzed protein data from the SGF, amygdala, and EC to determine which cytokines were most uniquely correlated with pathology found in the EC (Figs 3 and 4), the region most severely affected in AD. The EC contains long myelinated neurons that stem from layer II of the EC to form the perforant pathway^43^. These long neurons require increased axonal transport machinery and have greater metabolic requirements compared with neurons from other regions, which may make them more susceptible to homeostatic dysregulation and NFT and Aβ pathology^43^,^44^. Since glia respond to both NFTs and Aβ plaques, the differences we observed in the cytokine profiles between regions may reflect dysfunction of these sensitive neurons. Increased expression of neurotoxic cytokines in the EC, including TNF-α and IFN-γ (Fig. 4D), may contribute to neuronal stress through a positive feedback loop.

Despite its limitations, the ability of our PLSR profiling approach to detect cytokine drivers of AD was validated in that several of the signals correlated with ABC score have prior connections to the disease. For example, TNF-α has previously been linked to neuronal death and AD pathogenesis^17^,^34^, and our PLSR analysis identified it as a top correlate in the EC and the second strongest cytokine correlate in the ratio model (Figs 1c and 4a). Not surprisingly, TNF-α was a potent inducer of neuronal death in our neuron culture viability screen (Fig. 5a). While this type of validation is crucial, our ultimate goal is to use our protein profiling approach to suggest novel hypotheses about specific cytokines that may cause neuronal death in AD. For example, our ratio model identified VEGF and IL-5 as the first and third top cytokine correlates with ABC score (Fig. 4a), although neither has clearly been linked to promoting AD progression (Supplementary Results).

Our neuron culture viability screen was directed based on the hypotheses suggested by our cytokine profiling. The screen revealed that IL-5 was essentially inert (Fig. 5a). Since IL-5 has been shown to promote progenitor differentiation into neurons, (Supplementary Fig. S7)^45^, its up-regulation in human tissues may represent an attempt to compensate for neuronal death. VEGF, however, showed a synthetic death phenotype when combined with soluble Aβ (Fig. 5a,b). These data suggest for the first time a deleterious role for VEGF over-expression in the context of AD.

Ours is not the first analysis to report VEGF up-regulation in AD; it has previously been reported to be up-regulated both in the CNS^46^ and plasma^47^. VEGF up-regulation during AD pathogenesis could be explained by dysregulated vascular activation in AD^46^, although VEGF has been shown to be expressed by reactive astrocytes^48^. In addition to its well-known vascular functions, VEGF has been shown to be neurotrophic^21^, promote neurogenesis^22^ and neural patterning^49^, and to be neuroprotective after cerebral focal ischemia^50^. Together, the compiled literature seemingly suggests that VEGF should have a positive effect on neuronal survival in AD, and it has been suggested that VEGF therapy might be used to treat neurodegenerative diseases^51^. But while the above studies point to a neuroprotective role for VEGF, they were not conducted in the presence of physiologic levels of Aβ. We found that VEGF reduced neuronal viability only when applied together with Aβ1–42 (Fig. 5a,b), demonstrating that multiple variables found within the context of the human disease produce a synthetic phenotype. As such, VEGF exhibits a deleterious function that is highly context dependent.

VEGF is a member of the platelet-derived growth factor family that signals through receptor tyrosine kinases. In order to understand how VEGF promotes neuronal death downstream of its receptor, we produced a second PLSR model based on phospho-protein signals. We observed a broad signaling shift through pro-survival pathways in cells treated with both VEGF and Aβ1–42 (Fig. 5c), which is consistent with the synthetic death phenotype. This pro-death activity could be rescued by treating cells with a growth factor, BDNF, that stimulated pro-survival signaling independent of VEGFR (Fig. 5b and Fig. S6c). The question remains as to how VEGF and Aβ1–42 cooperate to affect downstream signal transduction in such a profound way. Aβ can interact with both VEGF^52^ and VEGFR2^53^ to inhibit VEGF signaling. Nevertheless, our data suggest that Aβ, rather than acting as a simply dominant negative on VEGF activity, produces an actively deleterious down-regulation of survival signaling. This model is supported by our observation that treatment of neurons with the VEGFR1/2 receptor inhibitor Axitnib can rescue the pro-death activity of VEGF and Aβ1–42 (Fig. 5b).

Together, our results identify VEGF as a previously unrecognized driver of neuronal death in AD, and demonstrate the importance of cellular and molecular context in specifying biological activity. Given that multiple anti-VEGF drugs are used clinically, VEGF may be an actionable therapeutic target for AD pathology. More generally, our study highlights the value of using primary tissue protein profiling together with screening approaches to identify new mechanisms driving AD pathogenesis.

## Methods

### Bio-Plex tissue analysis

Bio-Plex Tissue Analysis. Tissues were collected and cryopreserved at the Massachusetts Alzheimer Disease Research Center in accordance with guidelines approved by the Institutional Review Board at the Massachusetts General Hospital. All tissues were collected post-mortem from patients who had provided prior informed consent. A tissue subdivision was cut from each sample on dry ice, then, thawed, homogenized and lysed in Bio-Plex cell lysis kit (Bio-Rad) according to the recommended protocol with the addition of one cOmplete mini tablet per 5 mL of lysis buffer solution (Roche). Homogenized, lysed solutions were placed in microcentrifuge tubes, end-over-end rotated at 4 °C for a minimum of 10 min, and centrifuged for 10 min at 13.2 kRPM. Lysate supernatant was transferred to fresh tubes, and stored at -80°C. For Bio-Plex analysis, samples were thawed on ice, analyzed using a Pierce BCA (Thermo Scientific), and normalized in lysis buffer to 7.5 μg per 25 μL. Our optimization of Bio-Plex cytokine measurements of human brain tissues have shown that this concentration is in the center of a linear range of Bio-Plex signal vs. cytokine concentration. Samples were then analyzed according to kit protocols for the Bio-Plex human 27-plex and 21-plex kits (Bio-Rad), and Neurodegenerative Panel 4 kits (Milliplex). VEGF and IL-15 measurements in the amygdala and SFG suffered from high background in the 27-plex and were separately re-acquired. Samples for the same region on different plates were normalized to the standard provided with each kit. Panel 4 was further corrected for plate-to-plate variation by using a discrete-PLSR analysis against plate identity, and removing the first plate-correlated component of variation. All kits were read on the Bio-Plex 200 system (Bio-Rad). Since Bio-Plex kits do not include an internal loading control, 27-plex and 21-plex measurements were further normalized using a multivariate mathematical approach given by Eq. 3.

### Sample normalization

While samples were normalized to total protein using BCA, Bio-Plex kits did not include any internal loading controls. We therefore used a mathematical approach, exploiting the multidimensionality of the datasets, to further normalize the 27-plex and 21-plex data. For a dataset consisting of *n* samples and *m* measurements per sample, the dataset, 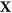, consisted of


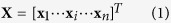


where 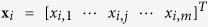 were the cytokine measurements collected from the *i*^th^ sample. Assuming that some of the cytokines were higher while others were lower in each of the control and disease classes, and assuming that the cytokines were in a linear range, we scaled all of the cytokines measured from a single sample using a single “loading” correction coefficient that minimized the sum-squared-error between each cytokine measurement 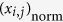 and the mean of that cytokine across the entire dataset, *μ*_*j*_. Then the loading correction coefficient for each sample *i* was computed as


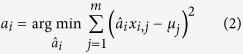


where 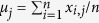 and the normalized dataset was


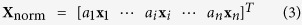


This method was separately applied to the 27-plex and 21-plex datasets, since each was measured in different wells on separate plates. To maintain consistent weighting for each cytokine in Eq. 2, each measurement, 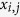, was normalized by 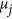 prior to computing the loading correction coefficients. This method was applied in MATLAB (Mathworks).

### Tissue gene expression analysis

Tissue subdivisions of approximately 5 mg were cut on dry ice from the same samples discussed for Bio-Plex analysis above. Subdivisions were lysed using a Quantigene sample processing kit (Affymetrix), then heated at 65 °C for 30 min while vortexing every 10min. Samples were then centrifuged for 15 min at 13.2 kRPM, and the supernatant was harvested. This step was repeated. Gene expression was analyzed using a Quantigene 2.0 (Affymetrix) plex set with probes for all of the cytokines used in the Bio-Plex kit and GAPDH. The Quantigene 2.0 assay was run according to kit protocol, then read out on a MAGPIX instrument (Luminex). Expression values were normalized to GAPDH and using Eq. 3.

### Partial least squares modeling

PLSR modeling was conducted in MATLAB using the partial least squares algorithm by Cleiton Nunes available on the Mathworks File Exchange. Bio-Plex cytokine sample data was pre-normalized as discussed above. All data was *z*-scored, and then directly inputted to the algorithm. For each PLSR analysis, an orthogonal rotation in the LV1-LV2 plane was used to choose a new LV1 that better separated phenotype/Y-variable. To avoid division-associated noise in the EC/SFG ratio analysis, a constant equal to the maximum signal value was added to each cytokine in the SFG dataset. Predictive modeling for LOOCV was conducted using the first two latent variables for each model. 95% confidence ellipsoids were computed using the inverse χ^2^ distribution with 2 degrees of freedom^54^. *p*-values for the slope of the ABC severity prediction curve for each LOOCV was computed using the regstats () t-statistic in MATLAB. To correct for sign reversals between models in the LOOCV, each sub-sampled LV1 and LV2 was multiplied by the sign of the scalar product of the new LV and the corresponding LV from the total model.

### Published gene expression data

The hippocampus CA1 gene expression dataset (Affymetrix Human Genome U133A Array) was downloaded from the NCBI Gene Expression Omnibus under accession number GSE 1297 (samples GSM21203-233). The gene identifiers were converted to Entrez Gene ID using the table for the microarray under accession number GPL96, and the individual genes corresponding to our Bio-Plex cytokine analysis were selected using MATLAB.

### Primary mouse neuron cultures

Neuron cultures were derived from E14–15 CD1 embryos (Charles River), according to a protocol approved by the Massachusetts General Hospital Institutional Animal Care and Use Committee. Embryo cortices were isolated according to an existing protocol^55^ and triturated in warm plating medium using a 1 mL pipette tip. Plating medium consisted of Neurobasal Medium (Invitrogen) with 10% FBS, 1x Glutamax (Gibco), and 1x antibiotic solution (Sigma). Cell concentration was measured using a hemocytometer, and cells were plated at 6750 cells/mm^2^ in poly-d-lysine (Sigma)-coated 96-well and 6-well plates for viability and Bio-Plex phospho-protein analysis, respectively. Cells were left to attach overnight in plating medium, then switched to neuron medium containing 1x B27 supplement (Invitrogen) instead of FBS. After 3 days *in vitro*, 5 μM 5-Fluoro-2-deoxyuridine (Sigma) was added to prohibit glial proliferation. Cultures were matured for a total of 9–11 days *in vitro*, then used for condition experiments. Conditions were applied together with a change of one-half of the medium in each well.

For viability studies, wells in 96-well plates were treated for 3 days with combinations of HFIP pre-treated recombinant human Aβ1–42 (50 nM; rPeptide, Bogart, GA) in 1% w/v NH_4_OH vehicle (final concentration of 0.001% NH_4_OH), Axitinib (1 μM; Selleckchem) and cytokines (100 ng/mL): TNF-α (Abazyme), VEGF164 (R&D), IL-12p70 (R&D), IL-5 (R&D), BDNF (R&D). Conditions were applied together with a change of one-half of the medium in each well. After 3 days, live/dead viability was assessed by staining with 1 μM Calcein AM (Invitrogen) and 2 μM Ethidium Homodimer (Invitrogen) for 15 mins. Wells were imaged using a 10x objective on a Nikon TE-2000S microscope with 1 second exposure time. Two locations were imaged at opposite sides of each well and treated as independent measurements. Each condition was applied to two separated rows in each plate to control for the effect of position on viability. For viability quantification, the background was first subtracted from all images in ImageJ (National Institutes of Health, USA) using the rolling ball algorithm with a radius of 800pixels. Subsequently, the images were imported into MATLAB. For the dead stain, the images were thresholded at 0.1 and number of spots was counted using the regionprops () function. For the live stain, all images were thresholded at 0.15 and the total area was summed. In each experiment, all values were normalized to the mean viability of an appropriate vehicle-treated condition. The Jarque-Bera test revealed that the viability data were not derived from a normal distribution. Therefore, we used the two-sided Wilcoxon Rank Sum test to assess significance.

For phospho-protein signaling studies, Aβ1–42 was pre-oligomerized overnight at 37 °C and 5% CO_2_. Vehicle was similarly incubated for non-Aβ1–42 wells. Subsequently, vehicle or Aβ1–42 was co-incubated at 37 °C and 5% CO_2_ with the cytokine (100 ng/mL) co-condition for 1 hr, then applied to 6-well plates together with a change of one-half of the medium. During the medium change, the re-used old medium was extracted from the wells, mixed, and centrifuged for 10 min at 5 kRPM to remove cell debris. After the treatment period, the cells were lysed using the same Bio-Plex lysis buffer used for tissue homogenates, as discussed above. Samples were normalized using the BCA assay and the Bio-Plex phospho-protein assay was conducted according to kit protocol with polystyrene beads for p-IκBα, p-MEK1, p-GSK3α/β, p-ATF-2, p-Stat3 (Y705), p-Akt, p-Jnk, p-Erk1/2, p-p38 MAPK, p-Stat3(S727) (Bio-Rad). Measurements from the BDNF culture experiment were normalized to the model-generating dataset using mean measurement values for each signal from (*N* = 3) wells harvested at 0 min. Since the control and VEGF conditions in the phospho-protein dataset for model generation represents the maximum realizable viabilities, the signaling measurements from the BDNF condition were thresholded to the maximum values obtained from the model-generating dataset prior to projection.

## Additional Information

**How to cite this article**: Wood, L. B. *et al.* Identification of neurotoxic cytokines by profiling Alzheimer's disease tissues and neuron culture viability screening. *Sci. Rep.* **5**, 16622; doi: 10.1038/srep16622 (2015).

## Supplementary Material

Supplementary Information
