# Supplementary material for: Identification of neurotoxic cytokines by profiling Alzheimer’s disease tissues and neuron culture viability screening
Source: Sci Rep. 2015 Nov 13;5:16622. doi: 10.1038/srep16622 (PMC4643219; doi:10.1038/srep16622)
Supplement: Supplementary Information [file srep16622-s1.pdf]

Identification of neurotoxic cytokines by profiling Alzheimer's disease tissues and neuron culture viability screening

Levi B. Wood[1], Ashley R. Winslow[2], Elizabeth A. Proctor[1,3], Declan McGuone[4,5], Daniel A. Mordes[4,5], Matthew P. Frosch[3,4,5], Bradley T. Hyman[3], Douglas A. Lauffenburger[3] & Kevin M. Haigis[1,*]

[1]Cancer Research Institute, Beth Israel Deaconess Cancer Center and Department of Medicine, Harvard Medical School, Boston, MA 02215, USA.

[2]Department of Neurology, Massachusetts General Hospital and Mass General Institute for Neurodegenerative Disease, Charlestown, MA 02129, USA.

[3]Department of Biological Engineering, Massachusetts Institute of Technology, Cambridge, MA 02139, USA.

[4]James Homer Wright Pathology Laboratories, Massachusetts General Hospital and Department of Pathology, Harvard Medical School, Charlestown, MA 02129, USA.

[5]C.S. Kubik Laboratory for Neuropathology, Massachusetts General Hospital and Department of Pathology, Harvard Medical School, Boston, MA 02114, USA.

*khaigis@bidmc.harvard.edu

Supplementary Information

Supplementary Results

Prior knowledge network reveals cytokines related to neuronal homeostasis

Our ultimate goal in profile the cytokine signaling network of the AD brain was to identify molecules that had not previously been connected to the disease, and therefore may represent novel therapeutic targets. Some of the molecules identified in our PLSR ratio model (*e.g.*, TNF-α) were linked to AD pathogenesis by prior studies[1]. In other cases, the connection between a given cytokine and AD was not as clear. To broadly place our analysis in the context of prior knowledge about the role of specific cytokines in the CNS, we surveyed the extensive prior work that investigated how cytokines influence phenotypic outcomes in CNS culture and animal models (**Supplementary Table S4**). An un-weighted spring-embedding network algorithm was employed to produce a description of the strength and diversity of these influences (**Supplementary Fig. S7**). The description illustrates that many of the cytokines identified in our PLSR model influence multiple aspects of the network, including Aβ levels, Tau hyper-phosphorylation (hp-Tau), and neuronal death. Because the prior knowledge network was constructed by minimizing overall edge length, cytokines with prior connections to AD and/or neuronal homeostasis (*e.g.*, TNF-α, IL-6, IL-10) are centrally located in the diagram (**Supplementary Fig. S7**). By contrast, cytokines that are more loosely connected to AD based on prior knowledge, including several identified in our PLSR model (*e.g.*, VEGF, IL-5), are peripherally localized in the prior knowledge network. We reasoned that these cytokines might represent potential new targets worthy of further investigation.

Supplementary Methods

Nodal interaction diagram

The interaction diagram, **Supplementary Fig. S7**, was generated using Cytoscape 3.1 (cytoscape.org). Nodes were created for each of the cytokines in our Bio-Plex panel that play a role in the CNS, and for each of the (color-coded) cell types expressing each cytokine. Additional nodes were added for phenotypic outcomes and for certain phospho-protein signaling events identified from the literature survey. Nodes were placed using a spring-embedding algorithm (Organic layout) to place connected nodes close together, and then manually- reconfigured so that all edges and nodes could be easily viewed.

Supplementary Figures and Tables

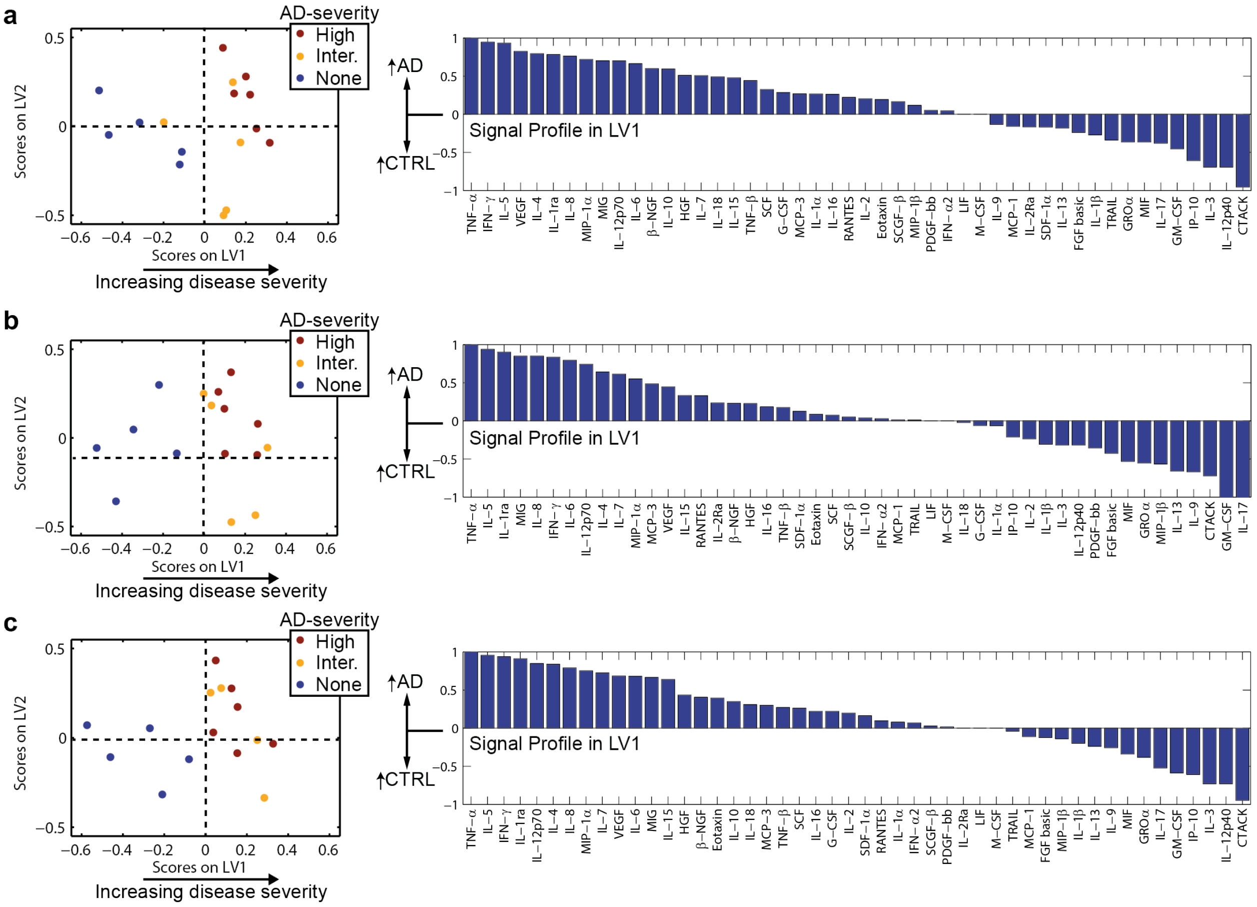

Supplementary Figure S1. Systems analysis of cytokines from AD and non-AD control subjects. PLS models constructed from the panel dataset regressed against (**a**) Thal-phase for Aβ plaques (A-score), (**b**) modified Braak and Braak stage (B-score), and (**c**) CERAD score for neuritic plaques (C-score).

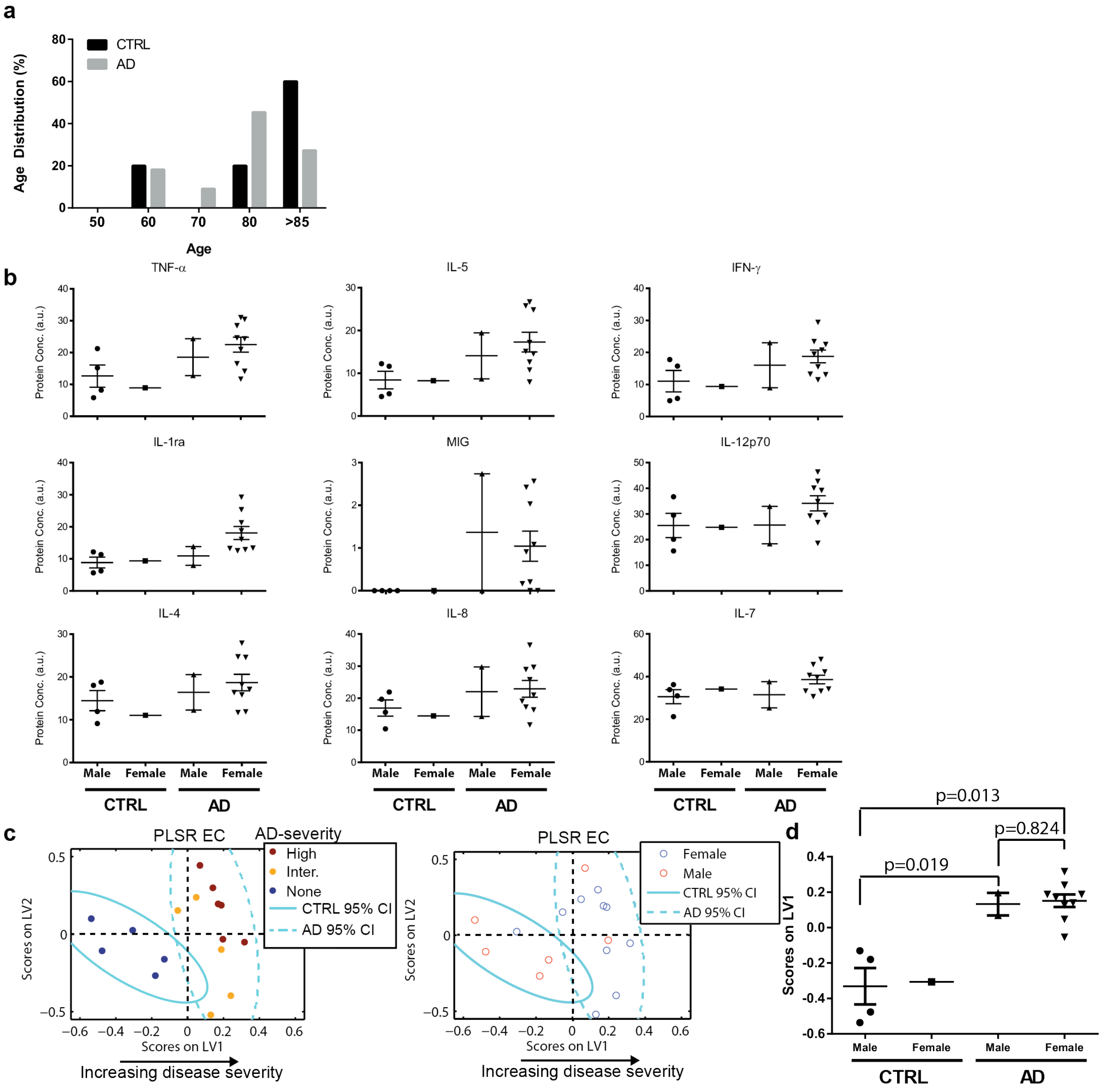

Supplementary Figure S2. Influence of age and gender differences between CTRL and AD groups on EC regression analysis. (**a**) Age distribution of CTRL and AD groups as a percentage of each group. (**b**) Male vs. female concentration of top 9 cytokines from LV1 of EC PLSR analysis (**Fig. 1c**). (**c**) Plotting samples in scores space from EC PLSR model and labeling for AD-severity and gender does not suggest a gender associated bias in the model space. Ellipses represent 95% confidence intervals for CTRL and AD groups. (**d**) Plotting of LV1 values from the EC model for each gender/disease group indicates that both male and female AD groups are significantly different from the male CTRL group. Further, the difference between male and female AD groups is insignificant (p-values were computed using a two-tailed t-test and were not corrected for multiple hypothesis testing).

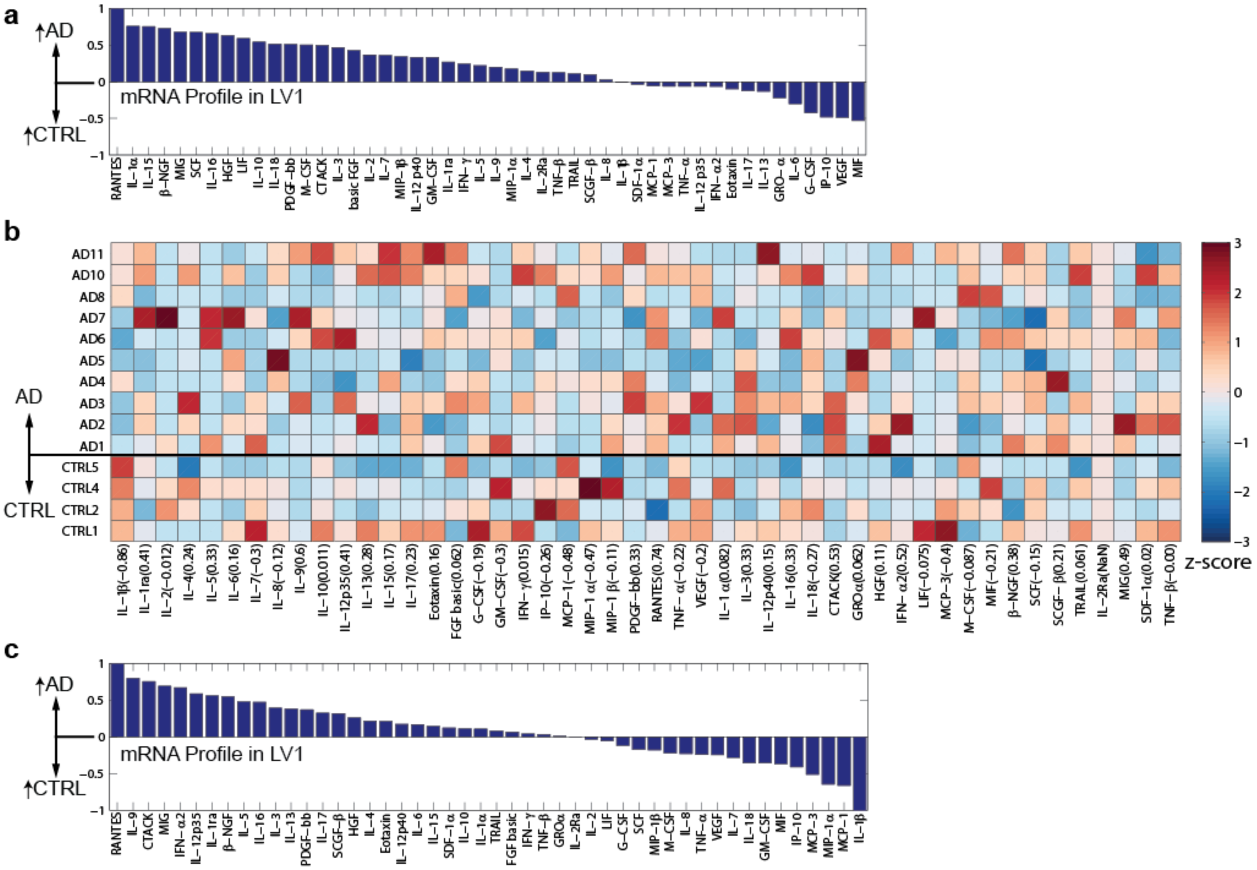

Supplementary Figure S3. Analysis of gene expression in the EC. (**a**) LV1 for PLSR analysis of published mRNA dataset (**Fig. 2a**) (**b**) Panel data for gene expression measured from subdivisions of the same EC samples used for Bio-Plex analysis. Note that we did not have sufficient tissue sample to analyze gene expression in CTRL3 and AD9. Values in parentheses are the Pearson's correlation coefficients relating each signal to ABC score. (**c**) LV1 for PLSR analysis of gene expression measured from subdivisions of the same EC samples used for Bio-Plex analysis **(Fig. 2b)**

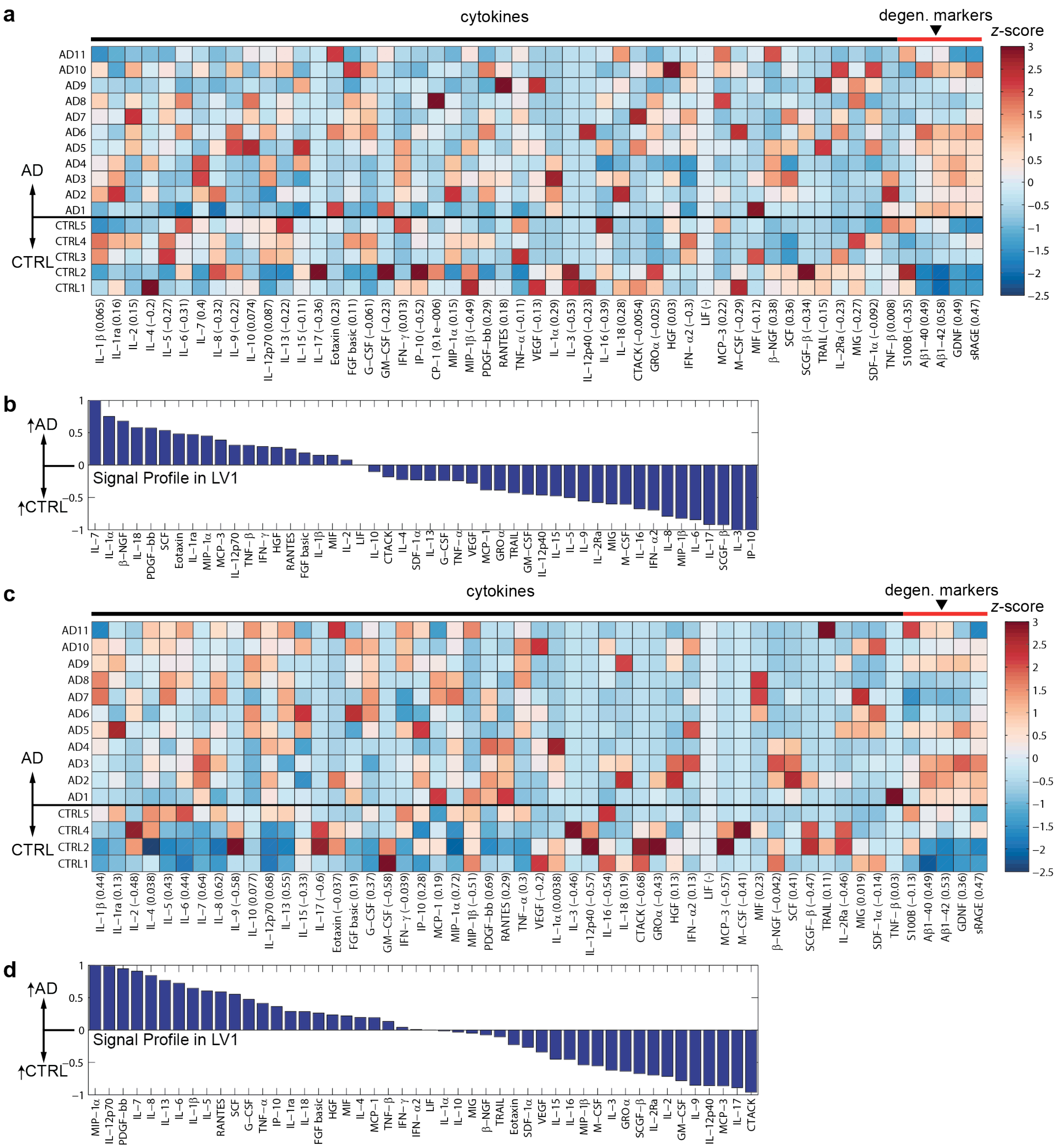

Supplementary Figure S4. Cytokine/neurodegeneration marker measurements and LV1 from postmortem SFG and amygdala tissues. (**a**) Heat map for superior frontal gyrus (SFG) samples. Values in parentheses are the Pearson's correlation coefficients relating each cytokine/signal to ABC score. (**b**) LV1 for PLSR model constructed by regressing the SFG cytokine values against the ABC scores. (**c**) Heat map for amygdala samples. Note that we did not have an amygdala tissue sample for CTRL3. Values in parentheses are the Pearson's correlation coefficients relating each signal to ABC score. (**d**) LV1 for PLSR model constructed by regressing the amygdala cytokine values against the ABC scores.

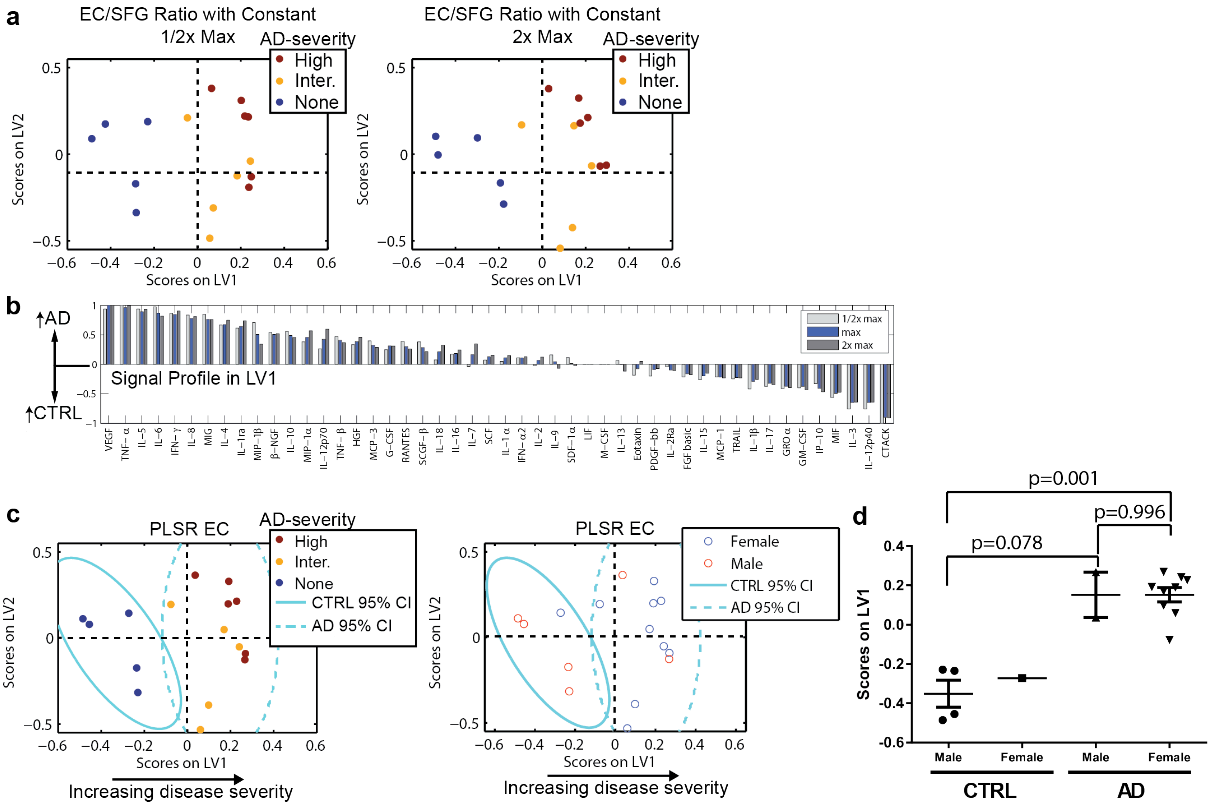

Supplementary Figure S5. Ratio regression model sensitivity to denominator constant and gender bias analysis. (**a**) Scores plots for EC/SFG models computed with a constant of 1/2x max or 2x max of each signal added to the SFG. (**b**) LV1 for the EC/SFG ratio model computed with a constant of 1/2x max, max, or 2x max of each signal added to the SFG. (**c**) Plotting samples in scores space from EC/SFG ratio PLSR model and labeling for AD-severity and gender does not suggest a gender bias in the model space. Ellipses represent 95% confidence intervals for CTRL and AD groups. (**d**) Plotting of LV1 values from the EC/SFG ratio model for each gender/disease group indicates that both male and female AD groups are different from the male CTRL group (though not at the p<0.05 significance threshold). Further, the difference between male and female AD groups is insignificant (p-values were computed using a two-tailed t-test and were not corrected for multiple hypothesis testing).

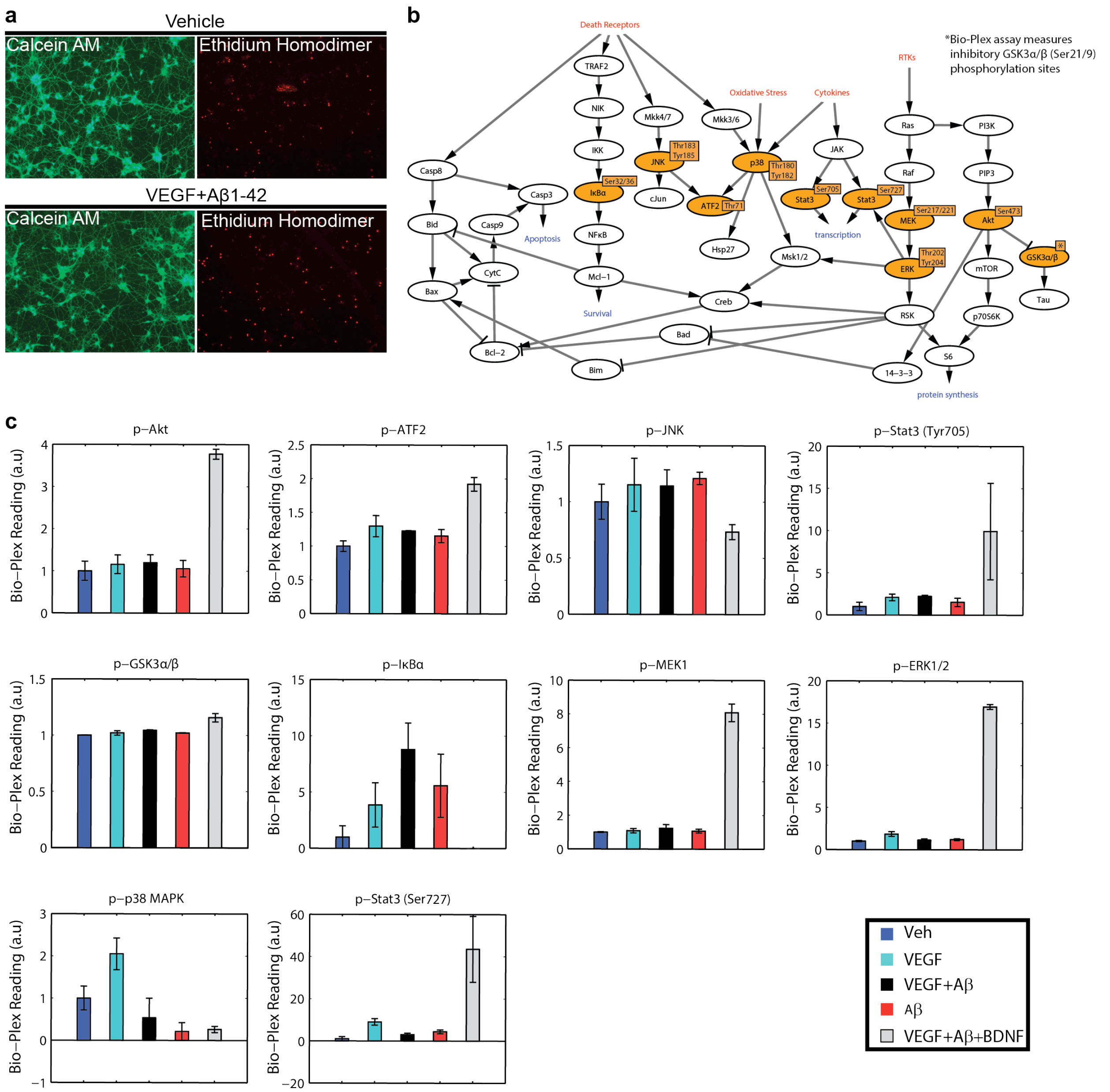

Supplementary Figure S6. Neuron viability and phospho-protein signaling analysis in primary mouse neurons. (**a**) Neuron viability was assessed after treating 9-11 day-old CD1 cultures for 3 days with either vehicle or 50nM Aβ1-42 and recombinant cytokines. Viability assessment was conducted by Live/Dead staining with Calcein AM/Ethidium homodimer (representative images shown). Images were quantified using ImageJ and MATLAB as detailed in **Methods**. (**b**) A broad collection of signaling nodes governing cell fate were measured via Bio-Plex (highlighted in orange) to identify mechanisms behind VEGF/Aβ induced cell death. Diagram edges were constructed via the Kyoto Encyclopedia of Genes and Genomes[2] and other references[3,4]. (**c**) Effect of BDNF on phospho-protein signaling 5min post-treatment (mean±SE, *N*=2-3).

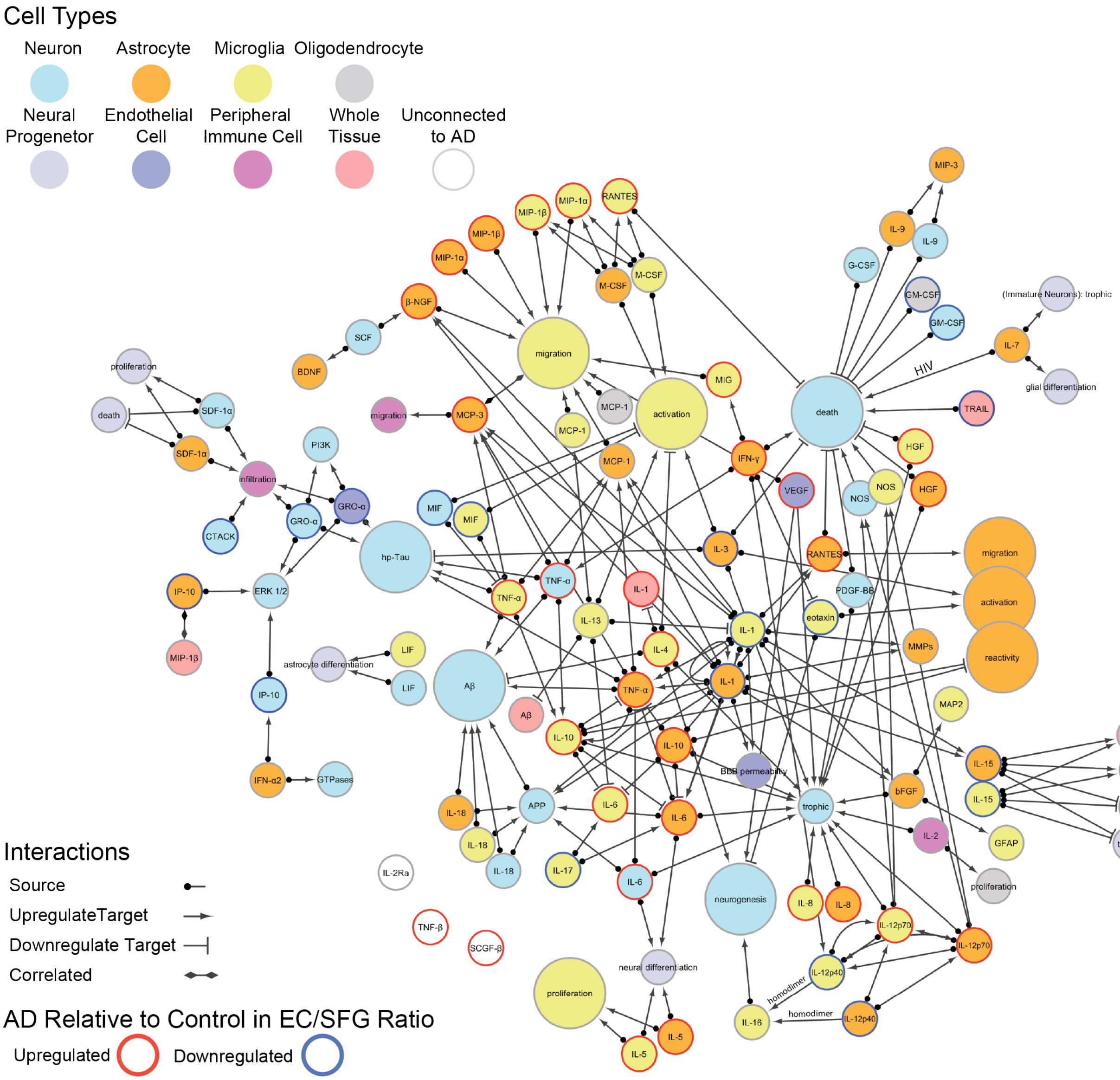

Supplementary Figure S7. Prior knowledge network of cytokine function in the CNS. Each node represents cytokine secretion or phenotypic outcomes for the color-coded cell type, and edges represent known interactions between the nodes. Cytokines expressed from multiple cell types appear multiple times with different-color codes. The network was initially generated using a spring-embedding algorithm to place connected nodes together and then manually re-positioned for clarity. Nodes enclosed with red rings denote the top 2/3 of positively correlated cytokines in the EC/SFG PLSR model, while nodes enclosed with blue rings indicate the top 2/3 of cytokines that are negatively correlated in the model. Nodes with gray rings were either not in to top 2/3 of positive and negative correlates, or were not measured. Phenotypic outcomes for neurons, astrocytes, and microglia are emphasized with larger nodes in the graph. Omissions for clarity: (1) IL-1ra is not shown, but competitively binds receptors for IL-1 and is not biologically active. (2) IL-1α and IL-1β are not distinguished. See **Supplementary Table S4** for specific information about each cytokine, including known mediators.

Supplementary Table S1. ABC-severity scoring for PLSR Regression[5].

**AD neuorpathalogic change**		**B**[b]		
**A**[a]	**C**[c]	**0 or 1**	**2**	**3**
**0**	**0**	none (0[d])	none (0[d])	none (0[d])
**1**	**0 or 1**	low (1[d])	low (1[d])	low (1[d])
	**2 or 3**	low (1[d])	intermediate (2[d])	intermediate (2[d])
**2**	**Any C**	low (1[d])	intermediate (2[d])	intermediate (2[d])
**3**	**0 or 1**	low (1[d])	intermediate (2[d])	intermediate (2[d])
	**2 or 3**	low (1[d])	intermediate (2[d])	high (3[d])

[a] Thal phase for Aβ plaques[6] translated to a scale from 0-3. **[b]**Braak & Braak stage for NFTs[7] translated to a scale from 0-3. **[c]**Consortium to Establish A Registry for Alzheimer's Disease (CERAD) neuritic plaque score[8] translated to a scale from 0-3. **[d]**Numerical ABC value assigned to "*Y*" variable for partial least squares regression.

Supplementary Table S2. Subject information.

Subject ID	Age of Death	Duration	Gender	A[a]	B[b]	C[c]	ABC-severity
Control Subjects							
CTRL 1	63	Control	M	0	0	0	none
CTRL 2	>89	Control	M	0	2	0	none
CTRL 3	>89	Control	F	0	1	1	none
CTRL 4	82	Control	M	0	1	0	none
CTRL 5	>89	Control	M	0	1	0	none
Alzheimer's Subjects							
AD1	59	7	F	2	3	3	intermediate
AD2	74	11	F	3	3	3	high
AD3	>89	8	F	3	3	3	high
AD4	62	9	F	3	3	3	high
AD5	>89	7	F	1	2	3	intermediate
AD6	79	15	F	2	3	3	intermediate
AD7	88.5	6.5	F	3	3	2	high
AD8	76	10	M	3	3	3	high
AD9	81	6	F	2	3	2	intermediate
AD10	79	2	F	1	3	3	intermediate
AD11	77	12	M	3	3	3	high

[a]Thal phase for Aβ plaques[6] translated to a scale from 0-3. [b]Braak & Braak stage for NFTs[7] translated to a scale from 0-3. [c]Consortium to Establish A Registry for Alzheimer's Disease (CERAD) neuritic plaque score[8] translated to a scale from 0-3.

Supplementary Table S3. Percent variance captured and LOOCV features for PLSR models fit to Bio-Plex and mRNA datasets. Fit parameters were computed by using a linear regression to of the predicted ABC score vs the true ABC score for each sample. The null hypothesis used for reporting the *p*-value is a slope = 0.

	**SFG**	**Amygdala**	**EC**	**EC/SFG Ratio**	**Published mRNA**	**EC mRNA**	**5 Signal Published mRNA**
Cytokine Variance Captured in Each LV							
% Var. in LV1	10.3	23.8	18.2	14.2	20.5	11.8	30.7
% Var. in LV2	13.3	11.4	16.1	17.3	8.7	16.1	26.8
LOOCV Fit Parameters							
Corr. Coeff, *R*	0.74	0.84	0.83	0.86	0.74	0.85	0.76*
p (2 LVs)	1.06×10^{-3}	1.00×10^{-5}	6.89x10	1.90×10^{-5}	2.14×10^{-6}	1.23×10^{-4}	1.50×10^{-3}*

*Computed for prediction of the ABC scores of our mRNA data based on the PLSR model produced from the Top 5/Bottom 5 signals in the published dataset.

Supplementary Table S4. Cytokine interaction prior knowledge.

Cytokine	Expression Location	Function in the CNS
IL-1β, IL-1α	Expressed primarily by microglia in CNS injury, directly related to Aβ plaque formation[9]. Positive feedback loop in microglia, astrocytes, and endothelial cells[10]. Up-regulated by APP[11].	Causes astrocyte proliferation and secretion of MMPs, IL-1, IL-6, TNF, NGF[12]. In endothelial cells, up-regulates E and P selectins, MCP-1, increased permeability of BBB[12]. Up-regulated APP, astrocyte proliferation and overexpression of S100B[9]. Up-regulated APP expression in PC12 cultures[13]. Up-regulated TNF-α expression in rat astrocyte cultures when applied together with IFN-γ[14]. Up-regulated RANTES expression in primary human astrocytes in the presence of IFN-β. IL-1β up-regulated MCP-1 and MCP-3 in cultured rat astrocytes via NFκB and p38/JNK pathways[15]. IL-1β up-regulated bFGF expression from astrocytes in rat brain[16].
IL-1ra	Astrocyote and microglia expressed in response to norepinephrine[17].	Receptor agonist of IL-1, anti-inflammatory[18].
IL-2	Up-regulated in peripheral mononuclear cells in moderate-severe AD[19].	Promoted survival and neurite extension in neuron culture, and stimulated proliferation of oligodendrocyte cultures[20].
IL-4	IL-4 expressed by rat microglia but not neurons or astrocytes *in vivo*[21]. IL-4 receptor expressed by astrocytes, neurons[22,23].	Reduced astrocyte/microglial activation, reduced Aβ deposition, and increased neurogenesis in APP/PS1 mouse model[22]. Provided trophic support for immature rat neuron cultures[24]. Down-regulated IL-1β in rat hippocampus[22].
IL-5	Ligand, but not receptor, is expressed by astrocytes and microglia[25].	Induced proliferation in rat microglial cultures[26]. Promoted Mk31 progenitor differentiation into neurons[27].
IL-6	Secreted by, astrocytes, and neurons[28-30] in response to IL-1β, TNF-α, IFN-γ. Secreted by microglia in response to pathogens and multiple cytokines[30]. Up-regulated in CSF of sporadic AD patients[31]. Down-regulated by estrogen and testosterone, and up-regulated with aging.	Up-regulated APP production in rat cortical neurons[11], and promoted neurite outgrowth. Enhanced cultured rat neuron survival under stress[30,32]. Enhanced neuronal differentiation from PC12 cultures in a process involving the STAT3 pathway[33]. Down-regulates TNF-α expression in rat astrocyte cultures[34].
IL-7	Astrocyte expressed[24]. Receptor, IL-7R, expressed by normal human neuron	Neuron apoptosis[36]. Mediated normal human neuron progenitor cell differentiation into astrocytes[35]. Provided trophic support for immature neuron

	progenitor cells[35].	cultures and increased number of astrocytes and microglia in culture[24].
IL-8	Astrocyte expressed[37]. Expressed by human microglial cultures treated with IFN-γ[38]. Projection neurons in the hippocampus, dentate nucleus and other locations express CXCR2 receptor, and Purkinje neurons in the cerebellum express Duffy Ag/receptor. Both receptors bind IL-8[37].	Provided trophic support for immature neuron cultures[24,37].
IL-9	Ligand and receptor primarily expressed by murine neurons *in vitro*[39]. Receptors, IL-9R and IL-2Rγ, were primarily expressed by astrocytes[40].	Inhibited neuron apoptosis in murine neocortex via JAK/STAT pathway[39]. Induced mouse astrocyte cultures to express MIP-3A[40]. Up-regulated Th17 cell migration into the mouse CNS[40].
IL-10	Secreted by microglia[41] and neonatal spinal cord astrocytes[42]. IL-10R expressed by cortical rat neurons[43].	Reduced IL-6 production in murine microglia cultures treated with LPS[44,45] by inhibiting NFκB activity. Protective of cortical rat neuron cultures via PI3K/AKT and STAT-3 pathways[43]. Inhibits TNF-α expression in mouse astrocyte cultures[34]. Shown to indirectly reduce astrocyte reactivity to injury[46]. Down-regulated IL-1α, IL-1β, TNF-α, and IL-6 in human microglia cultures treated with Aβ or LPS[45].
IL-12 (p70) IL-12 (p40)	IL-12p70 is a dimer incorporating IL-12p40 and IL-12p35 subunits, and IL-12p70 is typically considered to be the biologically active form[47]. IL-12 p40 expressed by microglia[12,48]. Both subunits expressed by astrocytes in LPS-treated murine astrocyte cultures[49]. IL-12p40 was also expressed by astrocytes in mouse brain treated with LPS[50]. though IL12p40 was not expressed in a murine AD model[51]. IL-12 receptor subunits β1 and β2expressed by	IL-12p40 up-regulated by IFN-γ and IL-12p70 in human microglial cultures[48]. Induced microglial expression of inducible nitric oxide synthase.[47] Up-regulated nitric oxide synthase in murine neuron cultures[52]. Promoted neurite outgrowth in mouse sympathetic ganglion neurons[53]. IL-12p40 homodimer induced expression of IL-16 in mouse and human microglia cultures[54].

	microglia[48]. Functional IL-12 receptor expression in murine neuron cultures[52].	
IL-13	Expressed by microglia, but not neurons or astrocytes of rat brain injected with LPS[55]. Expressed from rat microglia co-cultured with neurons, but not from microglia monoculture[55].	Down-regulated IL-1β and IL-6, but up-regulated MCP-1 secretion in human microglial cultures treated with Aβ or LPS[45]. Up-regulated microglial activation and Aβ clearance in 4.5 month old APP23 mice[56]. Sustained expression of inducible nitric oxide synthase and TNF-α, and increased microglia survival and neuronal death in rat cortex treated with both LPS and IL-13 neutralizing antibody[55].
IL-15	Expressed by astrocytes and microglia in human fetal cultures[57]. Receptor, IL-15Rα, is expressed by microglia in mouse brain[58], and by cultured rat neural stem cell and neurons, but not astrocytes or oligodendrocytes[59].	Increased expression from human fetal astrocyte cultures in response to IL-1β, IFN-γ, TNF-α, and in human fetal microglial cultures treated with INF-γ or LPS[57]. Up-regulated JAK1 pathway activity in mouse brain[58]. IL-15 knockout mice demonstrated memory deficit[60]. Reduced MAP-2 levels and neurite outgrowth, and up-regulated STAT3 phosphorylation in differentiating rat neuron cultures[59].
IL-17	Expressed by microglia in response to IL-1β and IL-2[61]. Receptor, IL-17R, expressed by mouse microglia and astrocytes, but not neurons.[61]	Works synergistically with IL-6 to induce IL-6 expression in primary astrocyte cultures, and involves NF-κB, JNK, and p38 pathway activation[62]. Up-regulated IL-6 production in mouse microglial cultures[61].
FGF basic	Expressed in astrocytes and some populations of neurons and microglia in rat brain[63]. Expressed from microglia, neurons, and most notably from astrocytes in rat cultures[64].	Chronic infusion of FGF basic after lesion was applied to rat EC enhanced survival of medial septal neurons[63]. Promotes generation of MAP2 positive and GFAP positive cells from rat microglial cultures[65]. Demonstrated to be necessary for adult hippocampal neurogenesis in mouse[66].
Eotaxin	Expressed by cultured murine microglia treated with pituitary adenylyl cyclase-activating polypeptide[67]. Receptors, CCR2, CCR3, CCR5 are expressed in human astrocytes[68].	Up-regulated cultured mouse astrocyte migration[68]. IP injection lead to inhibited neurogenesis in mice[69].
G-CSF	Ligand and receptor expressed by neurons in human and rat brains[70].	Reduced PARP and CC3 activity in cultured rat neurons treated with camptothecin NO[70].
GM-CSF	Ligand was expressed in rat neurons and	Anti-apoptotic affect in rat neuron cultures via PI3K-Akt pathway[71].

	oligodendrocytes while the receptor, GM-CSFR, was expressed only in rat neurons[71].	
IFN-γ	Expressed by adult rat astrocytes treated with TNF-α[72].	Up-regulated TNF-α expression in rat astrocyte cultures when applied together with IL-1β or LPS[14]. Co-cultures of primary rat neurons and microglia treated with IFN-γ and Aβ demonstrated neuronal loss, but not neuron monocultures treated with IFN-γ and Aβ[73]. Down-regulated eotaxin expression by murine microglial cultures[67]. Up-regulated MIG expression in mouse microglial cultures[74].
IP-10	Expressed in normal human brains and up-regulated in reactive astrocytes in AD brains[75]. Receptor, CXCR3, expressed in neurons[75].	Was up=regulated in Tg2576 mice[76]. Demonstrates coordinated up-regulation with MIP-1β in AD brains[75]. Up-regulates ERK1/2 in mouse neuron cultures[75].
MCP-1	Expressed by rat astrocytes and oligodendrocytes exposed to Aβ[77], and human fetal astrocytes and microglia treated with LPS[78].	Chemoattractant for rat and human microglial cultures[77-79].
MIP-1α	Expressed by human fetal astrocytes and microglia treated with LPS[78].	Was not changed in 9 month old Tg2576 mice[76]. Chemoattractant for human fetal microglia cultures[78].
MIP-1β	Expressed by human fetal astrocytes and microglia treated with LPS[78].	Chemoattractant for human fetal microglia cultures[78].
PDGF-BB	Expressed by neurons, but not other cell types in human brain[80]. Receptor expressed in rat brain neurons and cultured rat neurons[81].	Enhanced neurite outgrowth and neuron survival in primary rat cultures[81].
RANTES	Expressed by reactive astrocytes[77,82].	RANTES treatment of rat neuron cultures exposed to toxic thrombin and sodium nitroprusside enhanced survival[83]. Chemoattractant for rat microglial cultures[77].
TNF-α	Expressed by primary rat astrocyte cultures[34], human embryonic microglia cultures[29], and neurons in 3xTg-AD mice[84].	Up-regulated IL-10 expression by human fetal microglial cultures[85]. Focally administered TNF-α lead to up-regulated Aβ, tau hyper-phosphorlyation and microglial activation in 3xTg-AD mice[84]. Up-regulated MCP-3 expression by macaque astrocyte cultures[86]. Up-regulated MCP-1 and MCP-3 in cultured rat astrocytes via NFκB and p38/JNK

		pathways[15].
VEGF	Expressed by reactive astrocytes[87]. Receptor, Flt-1, expressed in human microglia[88].	Stimulated microglial chemotaxis in transwell, and receptor, Flt-1, is up-regulated in human AD samples[88]. Neurotrophic for cultured neurons[89] and shown to simulate neurogenesis[90].
IL-2Rα	-	-
IL-3	Expressed by cultured mouse astrocytes treated with endotoxin[91].	Activated mouse glial cultures via JAK2/STAT5 pathway[92]. Reduced tau phosphorylation, neurite loss and neuronal death in response to fibrillary Aβ via PI3K and JAK/STAT activation in primary mouse neuron cultures[93].
IL-16	Expressed by microglia but not astrocytes in murine and human cultures[54,94].	Expressed in areas of developing human brain that are undergoing neuropoesis[95].
IL-18	Expressed by neurons, purkinge cells, astrocytes and microglia[96]. Neuron-expressed receptors[96].	Up-regulated APP and APP cleavage via BACE-1[97] in SH-SY5Y cultures, resulting in increased Aβ production. Up-regulated GSK3β[97] and tau phosphorylation[98] in SH-SY5Y cultures.
CTACK	Expressed in mouse olefactory bulb neurons[99].	Anti-CTACK antibodies reduced the presence of T cells in mouse olfactory bulbs[99].
GRO-α	Expressed by neurons and endothelial cells in rat brain treated with soman (seizure model)[100]. Gro-α and receptor, CXCR2 were both expressed in activated microglia in human brain MS lesions[101].	Rat brain neuron and endothelial cell expression of GRO-α after soman treatment , followed by neutrophil infiltration[100]. Activated ERK1/2 and PI3K pathways, and up-regulated tau hyper-phosphorylation in mouse neurons[102].
HGF	Expressed in GFAP positive astrocytes, LN3 positive microglia, and occasional cortical neurons from human AD brains, and in the vicinity of Aβ plaques[103].	Neurotrophic effect on mouse dopaminergic neuron cultures[104]. Anti-apoptotic effect on neurons when administered to site of transient ischemia in gerbils[104].
IFN-α2	Gene expressed by GFAP positive astrocytes in mouse[105].	Up-regulated GTPases and IP-10 in mouse neuron cultures via STAT1 pathway[105].
LIF	Expressed by neurons in human brains[106] and activated rat microglial cultures[107].	LIF expressed by rat activated microglia promoted astrocyte differentiation from neural stem progenitor cells via JAK/STAT and MAPK activation[107].
MCP-3	Expressed by macaque astrocyte cultures[86].	Up-regulated migration of cultured macaque monocytes through a 3μm filter[86]. Correlated with macrophage and microglial migration in human gliomas[108].

M-CSF	Expressed by astrocytes during mouse brain development[109] and HIV-1 infected human fetal microglia[110]. Receptor, Fms tyrosine kinase expressed by microglia in rat brain[111].	Induced microglial activation involving PI3K, GTPase, and Rac activation in a microglial cell line[112]. Inducted MIP-1α and MIP-1β and up-regulated RANTES expression in human fetal microglia infected with HIV-1[110].
MIF	Basally expressed by hippocampal neurons[113] and expressed by microglia in the vicinity of Aβ plaques in APP mice[113].	Down-regulated microglial activation and microglial TNF-α expression in LPS treated mixed glia mouse cultures[114].
MIG	Expressed in mouse microglia, but not astrocyte, cultures[74]. Receptor, CXCR3, expressed in mouse and human microglia and astrocyte cultures[115].	Up-regulated migration in human microglial cultures[115].
β-NGF	Expressed by rat astrocyte cultures[116]. Receptor, TrkA, is expressed in mouse microglial cultures[117].	Chemotactically up-regulated rat microglial migration in a transwell[117].
SCF	Expressed mainly by neurons in mouse brain[118]. Expressed by mouse astrocytes during the first 7 days of culture and by cultured neurons and microglia[118].	Up-regulated mRNA of NGF, BDNF, ciliary neurotrophic factor in mouse astroglial cultures[118].
SCGF-β	-	-
SDF-1α	Expressed in human neurons and astrocytes[119]. Receptors, CXCR4 and CXCR7 are expressed by human neural progenitor cell[120] and activated rat microglial cultures[121].	Up-regulated human neuronal progenitor cell survival in culture via ERK1/2 activation[120]. Up-regulated microglial migration via ERK1/2 and proliferation via ERK1/2 and Akt signaling[121]. Disruption of SDF-1α polarization at the BBB correlated with leukocyte infiltration in MS[122].
TNF-β	-	-
TRAIL	Receptors, but not ligand, found in neurons, astrocytes, microglia of human brain[123].	Inducted apoptosis in rat neuron cultures[124].

Supplementary References

1 Tobinick, E., Gross, H., Weinberger, A. & Cohen, H. TNF-alpha modulation for treatment of Alzheimer's disease: a 6-month pilot study. *Med. Gen. Med.* **8**, 25 (2006).

2 Kanehisa, M. & Goto, S. KEGG: kyoto encyclopedia of genes and genomes. *Nucleic Acids Res* **28**, 27-30 (2000).

3 Berg, D., Holzmann, C. & Riess, O. 14-3-3 proteins in the nervous system. *Nat. Rev. Neurosci.* **4**, 752-762, doi:10.1038/nrn1197 (2003).

4 Force, T., Krause, D. S. & Van Etten, R. A. Molecular mechanisms of cardiotoxicity of tyrosine kinase inhibition. *Nat. Rev. Cancer* **7**, 332-344, doi:10.1038/nrc2106 (2007).

5 Montine, T. J. *et al.* National Institute on Aging-Alzheimer's Association guidelines for the neuropathologic assessment of Alzheimer's disease: a practical approach. *Acta. Neuropathol.* **123**, 1-11, doi:10.1007/s00401-011-0910-3 (2012).

6 Thal, D. R., Rub, U., Orantes, M. & Braak, H. Phases of A -deposition in the human brain and its relevance for the development of AD. *Neurology* **58**, 1791-1800, doi:10.1212/wnl.58.12.1791 (2002).

7 Braak, H. & Braak, E. Neuropathological stageing of Alzheimer-related changes. *Acta. Neuropathol.* **82**, 239-259 (1991).

8 Mirra, S. S. *et al.* The Consortium to Establish a Registry for Alzheimer's Disease (CERAD). Part II. Standardization of the neuropathologic assessment of Alzheimer's disease. *Neurology* **41**, 479-486 (1991).

9 Mrak, R. E. & Griffin, W. S. Interleukin-1, neuroinflammation, and Alzheimer's disease. *Neurobiol. Aging* **22**, 903-908 (2001).

10 Shaftel, S. S., Griffin, W. S. & O'Banion, M. K. The role of interleukin-1 in neuroinflammation and Alzheimer disease: an evolving perspective. *J. Neuroinflammation* **5**, 7, doi:10.1186/1742-2094-5-7 (2008).

11 Del Bo, R., Angeretti, N., Lucca, E., De Simoni, M. G. & Forloni, G. Reciprocal control of inflammatory cytokines, IL-1 and IL-6, and beta-amyloid production in cultures. *Neurosci. Lett.* **188**, 70-74 (1995).

12 Simi, A., Tsakiri, N., Wang, P. & Rothwell, N. J. Interleukin-1 and inflammatory neurodegeneration. *Biochem. Soc. Trans.* **35**, 1122-1126, doi:10.1042/BST0351122 (2007).

13 Chang, K. A. *et al.* Inhibition of the NGF and IL-1beta-induced expression of Alzheimer's amyloid precursor protein by antisense oligonucleotides. *J. Mol. Neurosci.* **12**, 69-74 (1999).

14 Chung, I. Y. & Benveniste, E. N. Tumor necrosis factor-alpha production by astrocytes. Induction by lipopolysaccharide, IFN-gamma, and IL-1 beta. *J. Immunol.* **144**, 2999-3007 (1990).

15 Thompson, W. L. & Van Eldik, L. J. Inflammatory cytokines stimulate the chemokines CCL2/MCP-1 and CCL7/MCP-3 through NFkB and MAPK dependent pathways in rat astrocytes [corrected]. *Brain Res.* **1287**, 47-57, doi:10.1016/j.brainres.2009.06.081 (2009).

16 Rivera, S., Gold, S. J. & Gall, C. M. Interleukin-1 beta increases basic fibroblast growth factor mRNA expression in adult rat brain and organotypic hippocampal cultures. *Brain Res. Mol. Brain Res.* **27**, 12-26 (1994).

17 McNamee, E. N., Ryan, K. M., Kilroy, D. & Connor, T. J. Noradrenaline induces IL-1ra and IL-1 type II receptor expression in primary glial cells and protects against IL-1beta-induced neurotoxicity. *Eur. J. Pharmacol.* **626**, 219-228, doi:10.1016/j.ejphar.2009.09.054 (2010).

18 Arend, W. P. The balance between IL-1 and IL-1Ra in disease. *Cytokine Growth Factor Rev.* **13**, 323-340 (2002).

19 Huberman, M., Sredni, B., Stern, L., Kott, E. & Shalit, F. IL-2 and IL-6 secretion in dementia: correlation with type and severity of disease. *J. Neurol. Sci.* **130**, 161-164 (1995).

20 Jiang, C. L. & Lu, C. L. Interleukin-2 and its effects in the central nervous system. *Biol. Signals Recept.* **7**, 148-156 (1998).

21 Park, K. W., Lee, D. Y., Joe, E. H., Kim, S. U. & Jin, B. K. Neuroprotective role of microglia expressing interleukin-4. *J. Neurosci. Res.* **81**, 397-402, doi:10.1002/jnr.20483 (2005).
22 Kiyota, T. *et al.* CNS expression of anti-inflammatory cytokine interleukin-4 attenuates Alzheimer's disease-like pathogenesis in APP+PS1 bigenic mice. *FASEB J.* **24**, 3093-3102, doi:10.1096/fj.10-155317 (2010).
23 Brodie, C., Goldreich, N., Haiman, T. & Kazimirsky, G. Functional IL-4 receptors on mouse astrocytes: IL-4 inhibits astrocyte activation and induces NGF secretion. *J. Neuroimmunol.* **81**, 20-30 (1998).
24 Araujo, D. M. & Cotman, C. W. Trophic effects of interleukin-4, -7 and -8 on hippocampal neuronal cultures: potential involvement of glial-derived factors. *Brain Res.* **600**, 49-55 (1993).
25 Sawada, M., Suzumura, A., Itoh, Y. & Marunouchi, T. Production of interleukin-5 by mouse astrocytes and microglia in culture. *Neurosci. Lett.* **155**, 175-178 (1993).
26 Liva, S. M. & de Vellis, J. IL-5 induces proliferation and activation of microglia via an unknown receptor. *Neurochem. Res.* **26**, 629-637 (2001).
27 Mehler, M. F., Rozental, R., Dougherty, M., Spray, D. C. & Kessler, J. A. Cytokine regulation of neuronal differentiation of hippocampal progenitor cells. *Nature* **362**, 62-65, doi:10.1038/362062a0 (1993).
28 Ershler, W. B. & Keller, E. T. Age-associated increased interleukin-6 gene expression, late-life diseases, and frailty. *Annu. Rev. Med.* **51**, 245-270, doi:10.1146/annurev.med.51.1.245 (2000).
29 Sebire, G. *et al.* In vitro production of IL-6, IL-1 beta, and tumor necrosis factor-alpha by human embryonic microglial and neural cells. *J. Immunol.* **150**, 1517-1523 (1993).
30 Gadient, R. A. & Otten, U. H. Interleukin-6 (IL-6)--a molecule with both beneficial and destructive potentials. *Prog. Neurobiol.* **52**, 379-390 (1997).
31 Blum-Degen, D. *et al.* Interleukin-1 beta and interleukin-6 are elevated in the cerebrospinal fluid of Alzheimer's and de novo Parkinson's disease patients. *Neurosci. Lett.* **202**, 17-20 (1995).
32 Yamada, M. & Hatanaka, H. Interleukin-6 protects cultured rat hippocampal neurons against glutamate-induced cell death. *Brain Res.* **643**, 173-180 (1994).
33 Wu, Y. Y. & Bradshaw, R. A. Synergistic induction of neurite outgrowth by nerve growth factor or epidermal growth factor and interleukin-6 in PC12 cells. *J. Biol. Chem.* **271**, 13033-13039 (1996).
34 Benveniste, E. N., Tang, L. P. & Law, R. M. Differential regulation of astrocyte TNF-alpha expression by the cytokines TGF-beta, IL-6 and IL-10. *Int. J. Dev. Neurosci.* **13**, 341-349 (1995).
35 Moors, M. *et al.* Interleukin-7 (IL-7) and IL-7 splice variants affect differentiation of human neural progenitor cells. *Genes Immun.* **11**, 11-20, doi:10.1038/gene.2009.77 (2010).
36 Nunnari, G. *et al.* Exogenous IL-7 induces Fas-mediated human neuronal apoptosis: potential effects during human immunodeficiency virus type 1 infection. *J. Neurovirol.* **11**, 319-328, doi:10.1080/13550280500187005 (2005).
37 Horuk, R. *et al.* Expression of chemokine receptors by subsets of neurons in the central nervous system. *J. Immunol.* **158**, 2882-2890 (1997).
38 D'Aversa, T. G., Eugenin, E. A. & Berman, J. W. CD40-CD40 ligand interactions in human microglia induce CXCL8 (interleukin-8) secretion by a mechanism dependent on activation of ERK1/2 and nuclear translocation of nuclear factor-kappaB (NFkappaB) and activator protein-1 (AP-1). *J. Neurosci. Res.* **86**, 630-639, doi:10.1002/jnr.21525 (2008).
39 Fontaine, R. H. *et al.* IL-9/IL-9 receptor signaling selectively protects cortical neurons against developmental apoptosis. *Cell Death Differ.* **15**, 1542-1552, doi:10.1038/cdd.2008.79 (2008).
40 Zhou, Y. *et al.* IL-9 promotes Th17 cell migration into the central nervous system via CC chemokine ligand-20 produced by astrocytes. *J. Immunol.* **186**, 4415-4421, doi:10.4049/jimmunol.1003307 (2011).
41 Aloisi, F., De Simone, R., Columba-Cabezas, S. & Levi, G. Opposite effects of interferon-gamma and prostaglandin E2 on tumor necrosis factor and interleukin-10 production in microglia: a

regulatory loop controlling microglia pro- and anti-inflammatory activities. *J. Neurosci. Res.* **56**, 571-580 (1999).

42 Werry, E. L., Liu, G. J., Lovelace, M. D., Nagarajah, R. & Bennett, M. R. Glutamate potentiates lipopolysaccharide-stimulated interleukin-10 release from neonatal rat spinal cord astrocytes. *Neuroscience* **207**, 12-24, doi:10.1016/j.neuroscience.2012.01.039 (2012).

43 Sharma, S. *et al.* IL-10 directly protects cortical neurons by activating PI-3 kinase and STAT-3 pathways. *Brain Res.* **1373**, 189-194, doi:10.1016/j.brainres.2010.11.096 (2011).

44 Heyen, J. R., Ye, S., Finck, B. N. & Johnson, R. W. Interleukin (IL)-10 inhibits IL-6 production in microglia by preventing activation of NF-kappaB. *Mol. Brain Res.* **77**, 138-147 (2000).

45 Szczepanik, A. M., Funes, S., Petko, W. & Ringheim, G. E. IL-4, IL-10 and IL-13 modulate A beta(1--42)-induced cytokine and chemokine production in primary murine microglia and a human monocyte cell line. *J. Neuroimmunol.* **113**, 49-62 (2001).

46 Balasingam, V. & Yong, V. W. Attenuation of astroglial reactivity by interleukin-10. *J. Neurosci.* **16**, 2945-2955 (1996).

47 Jana, M., Dasgupta, S., Pal, U. & Pahan, K. IL-12 p40 homodimer, the so-called biologically inactive molecule, induces nitric oxide synthase in microglia via IL-12R beta 1. *Glia* **57**, 1553-1565, doi:10.1002/glia.20869 (2009).

48 Taoufik, Y. *et al.* Human microglial cells express a functional IL-12 receptor and produce IL-12 following IL-12 stimulation. *Eur. J. Immunol.* **31**, 3228-3239, doi:10.1002/1521-4141(200111)31:11<3228::AID-IMMU3228>3.0.CO;2-7 (2001).

49 Constantinescu, C. S. *et al.* Astrocytes as antigen-presenting cells: expression of IL-12/IL-23. *J. Neurochem.* **95**, 331-340, doi:10.1111/j.1471-4159.2005.03368.x (2005).

50 Stalder, A. K. *et al.* Lipopolysaccharide-induced IL-12 expression in the central nervous system and cultured astrocytes and microglia. *J. Immunol.* **159**, 1344-1351 (1997).

51 Vom Berg, J. *et al.* Inhibition of IL-12/IL-23 signaling reduces Alzheimer's disease-like pathology and cognitive decline. *Nat. Med.* **18**, 1812-1819, doi:10.1038/nm.2965 (2012).

52 Ireland, D. D. & Reiss, C. S. Expression of IL-12 receptor by neurons. *Viral Immunol.* **17**, 411-422, doi:10.1089/0882824041856987 (2004).

53 Lin, H., Hikawa, N., Takenaka, T. & Ishikawa, Y. Interleukin-12 promotes neurite outgrowth in mouse sympathetic superior cervical ganglion neurons. *Neurosci. Lett.* **278**, 129-132 (2000).

54 Jana, M. & Pahan, K. IL-12 p40 homodimer, but not IL-12 p70, induces the expression of IL-16 in microglia and macrophages. *Mol. Immunol.* **46**, 773-783, doi:10.1016/j.molimm.2008.10.033 (2009).

55 Shin, W. H. *et al.* Microglia expressing interleukin-13 undergo cell death and contribute to neuronal survival in vivo. *Glia* **46**, 142-152, doi:10.1002/glia.10357 (2004).

56 Kawahara, K. *et al.* Intracerebral microinjection of interleukin-4/interleukin-13 reduces beta-amyloid accumulation in the ipsilateral side and improves cognitive deficits in young amyloid precursor protein 23 mice. *Neuroscience* **207**, 243-260, doi:10.1016/j.neuroscience.2012.01.049 (2012).

57 Lee, Y. B., Satoh, J., Walker, D. G. & Kim, S. U. Interleukin-15 gene expression in human astrocytes and microglia in culture. *Neuroreport* **7**, 1062-1066 (1996).

58 Hanisch, U. K. *et al.* Mouse brain microglia express interleukin-15 and its multimeric receptor complex functionally coupled to Janus kinase activity. *J. Biol. Chem.* **272**, 28853-28860 (1997).

59 Huang, Y. S. *et al.* Effects of interleukin-15 on neuronal differentiation of neural stem cells. *Brain Res.* **1304**, 38-48, doi:10.1016/j.brainres.2009.09.009 (2009).

60 He, Y. *et al.* Interleukin-15 receptor is essential to facilitate GABA transmission and hippocampal-dependent memory. *J. Neurosci.* **30**, 4725-4734, doi:10.1523/JNEUROSCI.6160-09.2010 (2010).

61 Kawanokuchi, J. *et al.* Production and functions of IL-17 in microglia. *J. Neuroimmunol.* **194**, 54-61, doi:10.1016/j.jneuroim.2007.11.006 (2008).

62 Ma, X. *et al.* IL-17 enhancement of the IL-6 signaling cascade in astrocytes. *J. Immunol.* **184**, 4898-4906, doi:10.4049/jimmunol.1000142 (2010).
63 Gomez-Pinilla, F., Lee, J. W. & Cotman, C. W. Basic FGF in adult rat brain: cellular distribution and response to entorhinal lesion and fimbria-fornix transection. *J. Neurosci.* **12**, 345-355 (1992).
64 Araujo, D. M. & Cotman, C. W. Basic FGF in astroglial, microglial, and neuronal cultures: characterization of binding sites and modulation of release by lymphokines and trophic factors. *J. Neurosci.* **12**, 1668-1678 (1992).
65 Niidome, T., Nonaka, H., Akaike, A., Kihara, T. & Sugimoto, H. Basic fibroblast growth factor promotes the generation of microtubule-associated protein 2-positive cells from microglia. *Biochem. Biophys. Res. Commun.* **390**, 1018-1022, doi:10.1016/j.bbrc.2009.10.100 (2009).
66 Cheng, Y., Black, I. B. & DiCicco-Bloom, E. Hippocampal granule neuron production and population size are regulated by levels of bFGF. *Eur. J. Neurosci.* **15**, 3-12 (2002).
67 Wainwright, D. A., Xin, J., Sanders, V. M. & Jones, K. J. Differential actions of pituitary adenylyl cyclase-activating polypeptide and interferon gamma on Th2- and Th1-associated chemokine expression in cultured murine microglia. *J. Neurodegen. Regen.* **1**, 31-34 (2008).
68 Dorf, M. E., Berman, M. A., Tanabe, S., Heesen, M. & Luo, Y. Astrocytes express functional chemokine receptors. *J. Neuroimmunol.* **111**, 109-121 (2000).
69 Villeda, S. A. *et al.* The ageing systemic milieu negatively regulates neurogenesis and cognitive function. *Nature* **477**, 90-94, doi:10.1038/nature10357 (2011).
70 Schneider, A. *et al.* The hematopoietic factor G-CSF is a neuronal ligand that counteracts programmed cell death and drives neurogenesis. *J. Clin. Invest.* **115**, 2083-2098, doi:10.1172/JCI23559 (2005).
71 Schabitz, W. R. *et al.* A neuroprotective function for the hematopoietic protein granulocyte-macrophage colony stimulating factor (GM-CSF). *J. Cereb. Blood Flow Metab.* **28**, 29-43, doi:10.1038/sj.jcbfm.9600496 (2008).
72 Xiao, B. G. & Link, H. IFN-gamma production of adult rat astrocytes triggered by TNF-alpha. *Neuroreport* **9**, 1487-1490 (1998).
73 Meda, L. *et al.* Activation of microglial cells by beta-amyloid protein and interferon-gamma. *Nature* **374**, 647-650, doi:10.1038/374647a0 (1995).
74 Ellis, S. L. *et al.* The cell-specific induction of CXC chemokine ligand 9 mediated by IFN-gamma in microglia of the central nervous system is determined by the myeloid transcription factor PU.1. *J. Immunol.* **185**, 1864-1877, doi:10.4049/jimmunol.1000900 (2010).
75 Xia, M. Q., Bacskai, B. J., Knowles, R. B., Qin, S. X. & Hyman, B. T. Expression of the chemokine receptor CXCR3 on neurons and the elevated expression of its ligand IP-10 in reactive astrocytes: in vitro ERK1/2 activation and role in Alzheimer's disease. *J. Neuroimmunol.* **108**, 227-235 (2000).
76 Duan, R. S. *et al.* Decreased fractalkine and increased IP-10 expression in aged brain of APP(swe) transgenic mice. *Neurochem. Res.* **33**, 1085-1089, doi:10.1007/s11064-007-9554-z (2008).
77 Johnstone, M., Gearing, A. J. & Miller, K. M. A central role for astrocytes in the inflammatory response to beta-amyloid; chemokines, cytokines and reactive oxygen species are produced. *J. Neuroimmunol.* **93**, 182-193 (1999).
78 Peterson, P. K., Hu, S., Salak-Johnson, J., Molitor, T. W. & Chao, C. C. Differential production of and migratory response to beta chemokines by human microglia and astrocytes. *J. Infect. Dis.* **175**, 478-481 (1997).
79 Cho, H. *et al.* Microfluidic Chemotaxis Platform for Differentiating the Roles of Soluble and Bound Amyloid-beta on Microglial Accumulation. *Sci. Rep.* **3**, 1823, doi:10.1038/srep01823 (2013).
80 Masliah, E., Mallory, M., Alford, M., Deteresa, R. & Saitoh, T. PDGF is associated with neuronal and glial alterations of Alzheimer's disease. *Neurobiol. Aging* **16**, 549-556 (1995).

81 Smits, A. *et al.* Neurotrophic activity of platelet-derived growth factor (PDGF): Rat neuronal cells possess functional PDGF beta-type receptors and respond to PDGF. *Proc. Natl. Acad. Sci. U. S. A.* **88**, 8159-8163 (1991).

82 Lee, H. P. *et al.* The expression of RANTES and chemokine receptors in the brains of scrapie-infected mice. *J. Neuroimmunol.* **158**, 26-33, doi:10.1016/j.jneuroim.2004.08.010 (2005).

83 Tripathy, D., Thirumangalakudi, L. & Grammas, P. RANTES upregulation in the Alzheimer's disease brain: a possible neuroprotective role. *Neurobiol. Aging* **31**, 8-16, doi:10.1016/j.neurobiolaging.2008.03.009 (2010).

84 Janelsins, M. C. *et al.* Chronic neuron-specific tumor necrosis factor-alpha expression enhances the local inflammatory environment ultimately leading to neuronal death in 3xTg-AD mice. *Am. J. Pathol.* **173**, 1768-1782, doi:10.2353/ajpath.2008.080528 (2008).

85 Hotta, K., Emala, C. W. & Hirshman, C. A. TNF-alpha upregulates Gialpha and Gqalpha protein expression and function in human airway smooth muscle cells. *Am. J. Physiol.* **276**, L405-411 (1999).

86 Renner, N. A., Ivey, N. S., Redmann, R. K., Lackner, A. A. & MacLean, A. G. MCP-3/CCL7 production by astrocytes: implications for SIV neuroinvasion and AIDS encephalitis. *J. Neurovirol.* **17**, 146-152, doi:10.1007/s13365-010-0017-y (2011).

87 Salhia, B. *et al.* Expression of vascular endothelial growth factor by reactive astrocytes and associated neoangiogenesis. *Brain Res.* **883**, 87-97, doi:http://dx.doi.org/10.1016/S0006-8993(00)02825-0 (2000).

88 Ryu, J. K., Cho, T., Choi, H. B., Wang, Y. T. & McLarnon, J. G. Microglial VEGF receptor response is an integral chemotactic component in Alzheimer's disease pathology. *J. Neurosci.* **29**, 3-13, doi:10.1523/JNEUROSCI.2888-08.2009 (2009).

89 Sanchez, A., Wadhwani, S. & Grammas, P. Multiple neurotrophic effects of VEGF on cultured neurons. *Neuropeptides* **44**, 323-331, doi:10.1016/j.npep.2010.04.002 (2010).

90 Jin, K. *et al.* Vascular endothelial growth factor (VEGF) stimulates neurogenesis in vitro and in vivo. *Proc. Natl. Acad. Sci. U. S. A.* **99**, 11946-11950, doi:10.1073/pnas.182296499 (2002).

91 Frei, K., Bodmer, S., Schwerdel, C. & Fontana, A. Astrocyte-derived interleukin 3 as a growth factor for microglia cells and peritoneal macrophages. *J. Immunol.* **137**, 3521-3527 (1986).

92 Natarajan, C., Sriram, S., Muthian, G. & Bright, J. J. Signaling through JAK2-STAT5 pathway is essential for IL-3-induced activation of microglia. *Glia* **45**, 188-196, doi:10.1002/glia.10316 (2004).

93 Zambrano, A., Otth, C., Maccioni, R. B. & Concha, II. IL-3 controls tau modifications and protects cortical neurons from neurodegeneration. *Curr. Alzheimer Res.* **7**, 615-624 (2010).

94 Zhao, M. L., Si, Q. & Lee, S. C. IL-16 expression in lymphocytes and microglia in HIV-1 encephalitis. *Neuropathol. Appl. Neurobiol.* **30**, 233-242, doi:10.1046/j.0305-1846.2003.00527.x (2004).

95 Schwab, J. M., Schluesener, H. J., Seid, K. & Meyermann, R. IL-16 is differentially expressed in the developing human fetal brain by microglial cells in zones of neuropoesis. *Int. J. Dev. Neurosci.* **19**, 93-100 (2001).

96 Alboni, S., Cervia, D., Sugama, S. & Conti, B. Interleukin 18 in the CNS. *J. Neuroinflamm.* **7**, 9, doi:10.1186/1742-2094-7-9 (2010).

97 Sutinen, E. M., Pirttila, T., Anderson, G., Salminen, A. & Ojala, J. O. Pro-inflammatory interleukin-18 increases Alzheimer's disease-associated amyloid-beta production in human neuron-like cells. *J. Neuroinflamm.* **9**, 199, doi:10.1186/1742-2094-9-199 (2012).

98 Ojala, J. O., Sutinen, E. M., Salminen, A. & Pirttila, T. Interleukin-18 increases expression of kinases involved in tau phosphorylation in SH-SY5Y neuroblastoma cells. *J. Neuroimmunol.* **205**, 86-93, doi:10.1016/j.jneuroim.2008.09.012 (2008).

99 Gunsolly, C. *et al.* Expression and regulation in the brain of the chemokine CCL27 gene locus. *J. Neuroimmunol.* **225**, 82-90, doi:10.1016/j.jneuroim.2010.04.019 (2010).

100 Johnson, E. A. *et al.* Increased expression of the chemokines CXCL1 and MIP-1alpha by resident brain cells precedes neutrophil infiltration in the brain following prolonged soman-induced status epilepticus in rats. *J. Neuroinflamm.* **8**, 41, doi:10.1186/1742-2094-8-41 (2011).

101 Filipovic, R., Jakovcevski, I. & Zecevic, N. GRO-alpha and CXCR2 in the human fetal brain and multiple sclerosis lesions. *Dev. Neurosci.* **25**, 279-290, doi:72275 (2003).

102 Xia, M. & Hyman, B. T. GROalpha/KC, a chemokine receptor CXCR2 ligand, can be a potent trigger for neuronal ERK1/2 and PI-3 kinase pathways and for tau hyperphosphorylation-a role in Alzheimer's disease? *J. Neuroimmunol.* **122**, 55-64 (2002).

103 Fenton, H. *et al.* Hepatocyte growth factor (HGF/SF) in Alzheimer's disease. *Brain Res.* **779**, 262-270 (1998).

104 Hamanoue, M. *et al.* Neurotrophic effect of hepatocyte growth factor on central nervous system neurons in vitro. *J. Neurosci. Res.* **43**, 554-564, doi:10.1002/(SICI)1097-4547(19960301)43:5<554::AID-JNR5>3.0.CO;2-H (1996).

105 Wang, J. & Campbell, I. L. Innate STAT1-dependent genomic response of neurons to the antiviral cytokine alpha interferon. *J. Virol.* **79**, 8295-8302, doi:10.1128/JVI.79.13.8295-8302.2005 (2005).

106 Rensink, A. A. *et al.* Expression of the cytokine leukemia inhibitory factor and pro-apoptotic insulin-like growth factor binding protein-3 in Alzheimer's disease. *Acta Neuropathol* **104**, 525-533, doi:10.1007/s00401-002-0585-x (2002).

107 Nakanishi, M. *et al.* Microglia-derived interleukin-6 and leukaemia inhibitory factor promote astrocytic differentiation of neural stem/progenitor cells. *Eur. J. Neurosci.* **25**, 649-658, doi:10.1111/j.1460-9568.2007.05309.x (2007).

108 Okada, M. *et al.* Tumor-associated macrophage/microglia infiltration in human gliomas is correlated with MCP-3, but not MCP-1. *Int. J. Oncol.* **34**, 1621-1627 (2009).

109 Thery, C., Hetier, E., Evrard, C. & Mallat, M. Expression of macrophage colony-stimulating factor gene in the mouse brain during development. *J. Neurosci. Res.* **26**, 129-133, doi:10.1002/jnr.490260117 (1990).

110 Si, Q., Cosenza, M., Zhao, M. L., Goldstein, H. & Lee, S. C. GM-CSF and M-CSF modulate beta-chemokine and HIV-1 expression in microglia. *Glia* **39**, 174-183, doi:10.1002/glia.10095 (2002).

111 Raivich, G., Gehrmann, J. & Kreutzberg, G. W. Increase of macrophage colony-stimulating factor and granulocyte-macrophage colony-stimulating factor receptors in the regenerating rat facial nucleus. *J. Neurosci. Res.* **30**, 682-686, doi:10.1002/jnr.490300412 (1991).

112 Imai, Y. & Kohsaka, S. Intracellular signaling in M-CSF-induced microglia activation: role of Iba1. *Glia* **40**, 164-174, doi:10.1002/glia.10149 (2002).

113 Bacher, M. *et al.* The role of macrophage migration inhibitory factor in Alzheimer's disease. *Mol. Med.* **16**, 116-121, doi:10.2119/molmed.2009.00123 (2010).

114 Rogove, A. D. & Tsirka, S. E. Neurotoxic responses by microglia elicited by excitotoxic injury in the mouse hippocampus. *Curr. Biol.* **8**, 19-25 (1998).

115 Biber, K. *et al.* Functional expression of CXCR3 in cultured mouse and human astrocytes and microglia. *Neuroscience* **112**, 487-497 (2002).

116 Park, M. H. *et al.* ERK-mediated production of neurotrophic factors by astrocytes promotes neuronal stem cell differentiation by erythropoietin. *Biochem. Biophys. Res. Commun.* **339**, 1021-1028, doi:10.1016/j.bbrc.2005.10.218 (2006).

117 De Simone, R., Ambrosini, E., Carnevale, D., Ajmone-Cat, M. A. & Minghetti, L. NGF promotes microglial migration through the activation of its high affinity receptor: modulation by TGF-beta. *J. Neuroimmunol.* **190**, 53-60, doi:10.1016/j.jneuroim.2007.07.020 (2007).

118 Zhang, S. C. & Fedoroff, S. Cellular localization of stem cell factor and c-kit receptor in the mouse nervous system. *J. Neurosci. Res.* **47**, 1-15 (1997).

119 Rostasy, K. *et al.* SDF-1alpha is expressed in astrocytes and neurons in the AIDS dementia complex: an in vivo and in vitro study. *J. Neuropathol. Exp. Neurol.* **62**, 617-626 (2003).

120 Zhu, B. *et al.* CXCL12 enhances human neural progenitor cell survival through a CXCR7- and CXCR4-mediated endocytotic signaling pathway. *Stem Cells* **30**, 2571-2583, doi:10.1002/stem.1239 (2012).
121 Lipfert, J., Odemis, V., Wagner, D. C., Boltze, J. & Engele, J. CXCR4 and CXCR7 form a functional receptor unit for SDF-1/CXCL12 in primary rodent microglia. *Neuropathol. Appl. Neurobiol.*, doi:10.1111/nan.12015 (2013).
122 McCandless, E. E. *et al.* Pathological expression of CXCL12 at the blood-brain barrier correlates with severity of multiple sclerosis. *Am. J. Pathol.* **172**, 799-808, doi:10.2353/ajpath.2008.070918 (2008).
123 Dorr, J. *et al.* Lack of tumor necrosis factor-related apoptosis-inducing ligand but presence of its receptors in the human brain. *J. Neurosci.* **22**, RC209 (2002).
124 Martin-Villalba, A. *et al.* CD95 ligand (Fas-L/APO-1L) and tumor necrosis factor-related apoptosis-inducing ligand mediate ischemia-induced apoptosis in neurons. *J. Neurosci.* **19**, 3809-3817 (1999).
